# Supplementary material for: Intervention fidelity and factors affecting the process of implementing a mobile phone text messaging intervention among adolescents living with HIV: a convergent mixed-methods study in southern Ethiopia
Source: BMJ Open. 2024 Jul 3;14(7):e077128. doi: 10.1136/bmjopen-2023-077128 (PMC11227767; doi:10.1136/bmjopen-2023-077128)
Supplement: Supplementary data [file bmjopen-2023-077128supp001.pdf]

Semistructured interview guide

Sociodemography of participants

S.no.	Age	Sex	Educational level	Caregiver/adolescent/healthcare provider	Hospital (site)

Adolescents

1. Are you receiving the mobile text-message reminder from the hospital?

Probe on:

- Do you remember the code through which you receive the text-message reminder?
- What would you like about the message?
- Is there any difficulty in the use of text terms for the time schedule or messaging frequency?

2. How did you find the text message reminder?

Probe:

•Benefits

•Challenges

3. What would you like to change or improve regarding the mobile text-messaging intervention?

4. For what other purposes do you use the mobile phone provided by the hospital?

5. Was there any technical problems regarding receiving text messages?

Probe: damage to devices, battery failure, text compatibility with phone,

6. How do you compare the text message reminder for medication against preintervention period?

Parents/caregivers:

1. How do family- and social-related factors affect ART adherence in adolescents?

2.What is the role of parents/caregivers in preventing adolescent antiretroviral treatment fatigue?

3.Have you heard about the text message intervention that your child receives?

4. How happy are you of the text-message reminder intervention?

5. Do you think that sending text-message reminders supports adherence?

6. Are the adolescent properly using the mobile phone provided from hospital? (probe: damage, stolen, used by other than the adolescent)

Health care providers

1. How did you experience the communication with the adolescents regarding this intervention?

2. Do you think this intervention improves the retention and adherence of adolescents?

3. Reporting damaged equipment

4. Benefits, challenges, and hope to integrate with the health care system?
