# Supplementary material for: Intervention fidelity and factors affecting the process of implementing a mobile phone text messaging intervention among adolescents living with HIV: a convergent mixed-methods study in southern Ethiopia
Source: BMJ Open. 2024 Jul 3;14(7):e077128. doi: 10.1136/bmjopen-2023-077128 (PMC11227767; doi:10.1136/bmjopen-2023-077128)
Supplement: Author's manuscript [file bmjopen-2023-077128.draft_revisions.pdf]

BMJ Open

BMJ Open is committed to open peer review. As part of this commitment we make the peer review history of every article we publish publicly available.

When an article is published we post the peer reviewers' comments and the authors' responses online. We also post the versions of the paper that were used during peer review. These are the versions that the peer review comments apply to.

The versions of the paper that follow are the versions that were submitted during the peer review process. They are not the versions of record or the final published versions. They should not be cited or distributed as the published version of this manuscript.

BMJ Open is an open access journal and the full, final, typeset and author-corrected version of record of the manuscript is available on our site with no access controls, subscription charges or pay-per-view fees (http://bmjopen.bmj.com).

If you have any questions on BMJ Open's open peer review process please email info.bmjopen@bmj.com

BMJ Open

Intervention fidelity and factors affecting the process of a mobile phone text messaging intervention among adolescents living with HIV: a convergent mixed methods study in southern Ethiopia

Journal:	*BMJ Open*
Manuscript ID	bmjopen-2023-077128
Article Type:	Original research
Date Submitted by the Author:	26-Jun-2023
Complete List of Authors:	Tunje, Abayneh; Lund University Faculty of Medicine, Department of Health Sciences; Arba Minch University, Public health<br>Persson, Helene ; Lund University Faculty of Medicine, Department of Health Sciences<br>Jerene, Degu; KNCV Tuberculosis Foundation,<br>Hallstrom, Inger; Lunds Universitet, Department of Health Sciences
Keywords:	Adolescent, HIV & AIDS < INFECTIOUS DISEASES, Information technology < BIOTECHNOLOGY & BIOINFORMATICS

SCHOLARONE™
Manuscripts

I, the Submitting Author has the right to grant and does grant on behalf of all authors of the Work (as defined in the below author licence), an exclusive licence and/or a non-exclusive licence for contributions from authors who are: i) UK Crown employees; ii) where BMJ has agreed a CC-BY licence shall apply, and/or iii) in accordance with the terms applicable for US Federal Government officers or employees acting as part of their official duties; on a worldwide, perpetual, irrevocable, royalty-free basis to BMJ Publishing Group Ltd ("BMJ") its licensees and where the relevant Journal is co-owned by BMJ to the co-owners of the Journal, to publish the Work in this journal and any other BMJ products and to exploit all rights, as set out in our licence.

The Submitting Author accepts and understands that any supply made under these terms is made by BMJ to the Submitting Author unless you are acting as an employee on behalf of your employer or a postgraduate student of an affiliated institution which is paying any applicable article publishing charge ("APC") for Open Access articles. Where the Submitting Author wishes to make the Work available on an Open Access basis (and intends to pay the relevant APC), the terms of reuse of such Open Access shall be governed by a Creative Commons licence – details of these licences and which Creative Commons licence will apply to this Work are set out in our licence referred to above.

Other than as permitted in any relevant BMJ Author's Self Archiving Policies, I confirm this Work has not been accepted for publication elsewhere, is not being considered for publication elsewhere and does not duplicate material already published. I confirm all authors consent to publication of this Work and authorise the granting of this licence.

For peer review only

Intervention fidelity and factors affecting the process of a mobile phone text messaging intervention among adolescents living with HIV: a convergent mixed methods study in southern Ethiopia

Abayneh Tunje, PhD-student[1,2*] abayneh_tunje.tanga@med.lu.se, Helene Åvik Persson, PHN, PhD[1] helene.avik_persson@med.lu.se, Degu Jerene, MD, PhD[1,2,3] degu.jerene.7501@med.lu.se, Inger Kristensson Hallström, RN, PhD, Professor[1] inger.kristensson_hallstrom@med.lu.se

Affiliations:

[1]Child and Family Health Unit, Department of Health Sciences, Faculty of Medicine, Lund University, Lund, Sweden.

[2] School of Public Health, College of Medicine and Health Sciences, Arba Minch University, Arba Minch, Ethiopia.

[3] KNCV Tuberculosis Foundation, The Hague, Netherlands.

Corresponding author:

*Corresponding author

Abayneh Tunje
abayneh_tunje.tanga@med.lu.se
The Faculty of Medicine
Department of Health Sciences
P.O. Box 157
221 00 Lund, Sweden

For peer review only

Abstract

Objective: To assess the intervention fidelity and explore contextual factors affecting the process of a mobile phone text messaging intervention in improving adherence to and retention in care among adolescents living with HIV, their families, and their healthcare providers in southern Ethiopia.

Design: A convergent mixed-methods design guided by the process evaluation theoretical framework and the RE-AIM framework was used alongside a randomised controlled trial to examine the fidelity and explore the experiences of participants in the intervention.

Setting: Six hospitals and five health centres providing HIV treatment and care to adolescents in five zones in southern Ethiopia.

Participants: Adolescents (aged 10–19), their families and their healthcare providers.

Intervention: Mobile phone text messages daily for 6 months or standard care (control).

Results: 306 participants were enrolled in the process evaluation. Among the intervention participants (N =153), 171 (55.9%) of whom were men, most resided in an urban area 225 (73.5%), and participants had a mean age of 15 (2.62). The overall experiences of implementing the text messages reminder intervention were described as helpful in terms of treatment support for adherence but had room for improvement. During the study, 30,700 text messages were sent, and fidelity was high, with 99.4% successfully receiving text messages during the intervention. Barriers such as failed text messages delivery, limitations in phone ownership, and technical limitations affected fidelity. Technical challenges can hinder maintenance, but a belief in the future of digital communication permeates the experiences of the text messages reminders.

For peer review only - http://bmjopen.bmj.com/site/about/guidelines.xhtml

Conclusions: Overall fidelity was high, and participants' overall experiences of mobile phone text messages were expressed as helpful. Contextual factors, such as local telecommunications networks and local electric power, as well as technical and individual factors must be considered when planning future interventions.

Trial registration: Pan African Clinical Trials **PACTR202107638293593, first registration** 20/07/2021.

Strengths and limitations with this study

- Secure and confidential delivery of high-volume messages through a 4-digit short code.
- The use of a mixed methods design and the inclusion of family members and healthcare providers is a strength of this process evaluation study.
- There was a limited number of adolescents´ family and healthcare providers included in the interviews.

For peer review only - http://bmjopen.bmj.com/site/about/guidelines.xhtml

INTRODUCTION

Globally, 38.4 million people were living with HIV in 2021. Of these, 1.71 million were adolescents aged 10–19, 88% of whom live in Sub-Saharan Africa (1, 2). To control the epidemic, the Joint United Nations Programme on HIV/AIDS (UNAIDS) launched the 95%–95%–95% targets aimed at HIV testing, treatment, and viral suppression by 2030 (3). However, by 2021, 75% percent of the 38.4 million people living with HIV (PLHIV) were receiving antiretroviral therapy (ART), a treatment gap of 5.9 million people (4). Moreover, achieving the recommended optimal adherence level of >95% which is required for ART to be effective, remains a challenge within the HIV care continuum (5). This poses a significant threat to HIV interventions in resource-limited settings (6).

Poor adherence to ART has several consequences, including increased risk of viral drug resistance and reduced treatment effectiveness toward viral suppression, leading to disease progression, greater risk of death, and increased risk of viral transmission (7). Some of the major barriers to ART adherence include socioeconomic status,(8, 9) fear of being stigmatised or discriminated against as a result of one's HIV status,(10) forgetting to take medication on time,(11) treatment fatigue,(12) and patient–provider communication (13). Several interventions to overcome these barriers have been studied in a variety of settings (14). The use of digital health strategies to enhance HIV treatment is one of the priority interventions in Sub-Saharan Africa (15). Targeted digital client communication using mobile phone-based short text messaging has been recommended for adherence support for a range of health issues and adherence to antiretroviral treatment (16). The success and effectiveness of an mHealth intervention in achieving desired health outcomes is dependent on the context in which they are implemented (15, 16). Context plays a crucial role in either the success or failure of an intervention. The same intervention may have

For peer review only - http://bmjopen.bmj.com/site/about/guidelines.xhtml

different effects in different contexts, due to weaknesses in its design or improper implementation (17). Observing what is delivered in practice with close reference to the theory of the intervention can help evaluators in distinguishing the intervention fit in different contexts and changes that undermine intervention fidelity (15, 17). Trials should continue to rigorously assess outcomes, but also include integral process evaluations that use qualitative and quantitative data to develop and test hypotheses about how interventions work (18). In a multi-centre trial, a process evaluation is necessary to understand whether the intervention was implemented and received similarly across all sites (9). However, trials assessing mHealth adherence interventions for HIV often do not include process evaluations to examine the fidelity and quality of the intervention delivery, causal mechanisms for the health outcomes, contextual factors affecting the delivery, and costs of implementation (16, 19). Understanding the entire implementation process of an mHealth intervention allows practitioners to interpret the results and replicate the intervention in other contexts (20, 21). Therefore, this study aimed to evaluate the process of a mobile text messaging intervention conducted among adolescents living with HIV in Ethiopia. This information can be used to explain the outcomes of the trial and can guide the development and evaluation of similar interventions.

For peer review only - http://bmjopen.bmj.com/site/about/guidelines.xhtml

METHODS

Study design

A convergent mixed-methods design (17) combining quantitative and qualitative techniques was used. The design was guided by the process evaluation theoretical framework (22) and structured by the RE-AIM dimensions (Reach, Effectiveness, Adoption, Implementation and Maintenance) (18) to evaluate intervention effectiveness alongside a randomised controlled trial (RCT) to examine the study's fidelity and to explore the experiences of the participants in the intervention.

The trial was registered in Pan African Clinical Trials Registry with the registration number PACTR202107638293593 on 17/07/2021.

Study setting and participants

Six hospitals and five health centres that provide HIV treatment and care to adolescents in the Gamo, Gofa, Konso, South Omo, and Wolayita zones in Southern Ethiopia were included in the study. These zones are home to over 25 distinct ethnic groups, each with its own culture and context. Between July 5, 2022, and February 28, 2023, 306 adolescents living with HIV were included in the study, in either the intervention or in the control group. The quantitative data included process indicators, while the qualitative interviews included ten adolescents from the main trial, four adolescents' families, and two healthcare providers who were involved in the process evaluation.

For peer review only - http://bmjopen.bmj.com/site/about/guidelines.xhtml

Theoretical Framework of Process Evaluation

The RE-AIM framework (18) was used for the process evaluation to comprehensively examine the level of implementation. This involves understanding the implementation of interventions in terms of (1) fidelity, defined as to what extent the intervention was implemented consistently with the underlying theory as planned; (2) delivery, defined as to what extent all of the intended activities, training, and materials were provided to program participants; (3) reach, defined as the absolute number, proportion, and representativeness of individuals who are willing to participate in a given initiative, intervention, or program; (4) receipt, defined as how participants reacted to specific aspects of the intervention; and (5) context, defined as what contextual factors influence implementation or the intervention outcome.

Description of the intervention

The MRC framework,(22) a systematic literature review,(23) interviews with adolescents,(24) and the theoretical health belief model (HBM) (25) were used to develop the intervention. Adolescents living with HIV, healthcare providers, and research project technical advisory team members participated in the design (26) under the guidance of a research advisory committee. The HBM is a theoretical model that has been constructed from six domains: susceptibility, severity, barriers, benefit, cues to action, and self-efficacy. After the intervention's development, text messages were uploaded to the server and an automated message-pushing software was used to push tailored daily text messages through a four-digit code obtained from Ethiopia Telecom company. Other various configurations such as wide-area network (WAN) internet protocol (IP), local area network (LAN) IP, Ethio telecom central server IP, a virtual private network (VPN) line, and office servers were used as infrastructure, as required to support the study. The text messages were categorised and

For peer review only - http://bmjopen.bmj.com/site/about/guidelines.xhtml

delivered based on each adolescent's customised medication schedule and time. The software sent the message 15 minutes before each participant’s medication time. The server was checked daily to troubleshoot network/cell phone issues. Based on the server information, the first author contacted the facility level care provider to contact the adolescent with corrective actions; for example, subscriber identity module card (SIM card) replacement, damaged mobile phones, and network troubleshooting for maintenance and replacement.

Sample and data collection

Related to the intervention, activity logs (checked by researchers and the advisory committee), adverse events related to the study (monitored by care providers at each facility through an adverse event monitoring form), and routine records of sent and failed messages from the server were assessed by researchers. Each facility representative assessed any adverse event related to study, filled in the form, and sent it to researchers every week, and the researcher tracks the related server information related with sent/failed messages daily.

A purposive sampling of 10 adolescents with HIV (aged 12–19), four family members/relatives (aged 36–45), and two healthcare providers (aged 31–36) from different healthcare facilities were asked to be interviewed about their experiences in the intervention between June and July 2022 at healthcare facilities by the first author (AT), and one colleague in the research advisory committee. An interview guide was used to gain a rich, in-depth understanding, and to allow the participants to concretise their experiences of their use of text messages, communication, and technical aspects of the intervention. The interview guide included subsections adapted to the participants; i.e., adolescents, families, and healthcare providers. Hence, the questions were different, depending on to whom they were addressed. The introductory question to the adolescents was: “Are you receiving the mobile text-message reminder from the hospital?”, followed by follow-up questions

For peer review only - http://bmjopen.bmj.com/site/about/guidelines.xhtml

such as, “What do you think about the messages?” The families were initially asked “How do family/social related factors affect ART adherence among adolescents?” Healthcare providers were asked “How did you experience communication with the adolescents with regard to the intervention?” Follow-up questions and prompts were used throughout the whole interview. The interviews were audiotaped, transcribed verbatim, and translated from Amharic to English by the first author, and checked for accuracy by one independent language expert.

Data analysis

The number of SMS messages sent and delivered to participants were tracked as a measure of intervention fidelity. When SMS messages did not arrive on the phone, the project advisory team investigated the external network and other infrastructure issues. If the message could not be delivered during this period, the computer automatically recorded it as a delivery failure and sent the message again. Data captured on the delivery status of the SMS messages were recorded as: delivered (the phone had reception marked as green); undelivered (the phone was switched off or marked as red in the server). The program could not record whether messages delivered to the phones were opened.

Interviews were analysed by using the principles for content analysis described by Graneheim and Lundman (19, 27). First, the transcripts were read several times to gain a sense of the whole by the first and the last author. Thereafter, meaning units were identified and condensed to reduce the text while maintaining the core meaning. Condensed meaning units were then labelled with a code, which were kept close to the text on a manifest level. Next, sub-themes were created from the code frame by the first and last authors. The sub-themes were then abstracted into themes. Finally, the underlying meaning—the latent content—was described into a main theme. Thereafter one of the

For peer review only - http://bmjopen.bmj.com/site/about/guidelines.xhtml

authors (HÅP) read the transcripts, extracted meaning units, codes, and themes. The content was discussed and reflected upon together with all authors and lasted until an agreement was reached.

RESULTS

The results are structured using the Reach, Effectiveness, Adoption, Implementation and Maintenance dimensions of the RE-AIM framework (18) and corresponding themes (Table 1).

Table 1. Overview of the qualitative themes related to the dimensions in the RE-AIM framework.

**RE-AIM dimension (18)**	**Definition (18)**	**Qualitative theme**
Reach	The number, proportion, and representativeness of eligible individuals who participate in each initiative.	
Effectiveness	The impact of an intervention on the relevant outcomes, including potential adverse effects, quality of life, and economic outcomes.	Text message reminders were helpful for adherence but there was room for improvement
		Text messages reminders increase medication adherence.
		Feeling comfortable receiving text messages reminders
		The code delivered with text messages reminders minimises the risk of stigma.
Adoption	The reach and effectiveness/efficacy of an intervention at the setting level.	Treatment support
Implementation	The intervention agents' fidelity to the various elements of an intervention's protocol, including consistency of intended delivery.	Failing text messages delivery influences medication intake
		Limitations in phone ownership
		Technical limitations
Maintenance	Connects with both setting-level indicators (the extent to which a program or a policy becomes part	Seeing potential in SMS services

For peer review only - http://bmjopen.bmj.com/site/about/guidelines.xhtml

	of organisational practices and policies), and individual-level indicators (monitoring of effectiveness of an intervention or program six months or more after the most recent contact).	Possibilities of text messages reminders

Reach:

The total number of individuals that were assessed for eligibility was 435, of which 306 (70.34 %) meet eligibility criteria. Thus, 306 participants were enrolled and randomised: n =153 in the intervention, and n =153 in the control group. Among the 306 enrolled participants, 171 (55.88%) were men, and 135 (44.12%) were women, respectively. The mean and standard deviation age of the participants is 15 (2.62). Of these, 225 (73.53%) resided in an urban area, 190 (62.3%) had primary school level education, and only 31(9.9 %) had owned mobile phones during recruitment.

Effectiveness:

The number of participants retained in the 6-month follow-up of the intervention group was higher than the control group in this study.

Text messages reminders were helpful for adherence but there was room for improvement

The overall experiences of the intervention among adolescents living with HIV was that text messages reminders were helpful when it came to their adherence to medication, but direct access to phones and better timing of messages should be assured to increase their functioning and usability. The retention rate was improved, and was the best achieved since the intervention was introduced. Despite this, more effort was requested concerning technical issues to make the

For peer review only - http://bmjopen.bmj.com/site/about/guidelines.xhtml

reminders even more helpful. The participants described facilitating aspects like increased medication intake and communication. Also, the well-being of the adolescents and the support function was highlighted. However, technical problems like lost telephones or text messages reminders arriving after medication time were emphasised as barriers.

Text messages reminders increase medication adherence.

The adolescents felt that the text messages reminders had helped them to increase adherence to their medication so that it was taken at the right time. The adolescents described that before the intervention, their medication was often forgotten, which resulted in it being taken later than it should have been due to forgetfulness. Sometimes the adolescent realised that they had forgotten to take their medication but then waited until dinner to take it. The adolescents described that text messages reminders were associated with taking their medicine and delays were avoided. Also, family members described situations when the adolescent's medication routines were interrupted, and where text messages reminders played a big role.

> *"…Yes, it supports treatment adherence, because most of time we follow the television program but sometimes the power goes off, we missed the exact time to see medication time. Currently this text message comes 10 to 15 minutes before our medication time." (Father, aged 45)*

Feeling comfortable receiving text message reminders

The text messages reminders contributed to positive attitudes and to decreased stigmatisation for adolescents, even when HIV/AIDS stigma is deeply rooted in the culture. Thus, the perception of

For peer review only - http://bmjopen.bmj.com/site/about/guidelines.xhtml

the adolescents was that the text messages reminders played an important role for their overall well-being, because sometimes they had low moods. The text messages reminders were described as offering hope and supported the adolescents in achieving consistency in their medication adherence which helped them avoid, for example, fatigue.

> *"…The benefit of this message is it comes always and reminds me to take the drug on time. The other benefit is when I receive the message, I feel good." (Adolescent, aged 13)*

The code-delivered text messages reminders minimises the risk of stigma

The adolescent felt safe regarding privacy in the text messages reminders due to a code sent out to their telephone. The healthcare provider noticed a change in the views of both adolescents and family regarding increased trust and improved engagement with healthcare facilities during the intervention. Family expressed receiving coded text messages reminders could enable adolescents to live like other HIV-free adolescents. However, the adolescents experienced that far from everyone talked openly about their HIV disclosure, which was also reflected in the interviews. Some members of the families were open with their HIV disclosure while others were not.

> *"My status has already been made public, and everybody knows about my family as well ... However, my husband lives in another area because of work, and knows his HIV status and makes things secret. He did not even take his medication when he come back home." (Mother, aged 45)*

Adoption:

In this study, eleven health facilities with 153 participants received an intervention across 5 different geographic areas. The healthcare workers expressed that the intervention's retention rate

For peer review only

For peer review only - http://bmjopen.bmj.com/site/about/guidelines.xhtml

was the best that had been achieved since the introduction of this study to the hospital, which is listed under a sub-theme in the qualitative findings.

Treatment support

The healthcare providers emphasised that text messages reminders were an important support for adolescents in their medication intake to avoid poor adherence. In addition to this, families indicated that they had a mission to support the adolescents in avoiding ART treatment fatigue. Several adolescents expressed that they had received support from their family concerning medication intake. Thus, the text messages reminders were perceived as helpful, as they made participants feel happy and hopeful, even if they felt angered or were in a depressed mood that day.

> *"…The messages may help people pass good days, despite days that do not carry good fortune. It therefore helps people have good days and reminds them of medication times. One day, for example, being consumed with some ideas, I did not recall that I have medication to take; it is this text message that triggered me take my medicine." (Adolescent, aged 19)*

Implementation:

There were 306 mobile phones provided to adolescents during assignments into intervention and control groups. The advisory committee was involved in the implementation of the intervention using standard operational procedure (SOP). During the study, 30,700 SMS text messages were sent to 153 study participants in the intervention group (Figure 1).

Overall, intervention fidelity was high with (30,510/30,700) or 99.38% successfully received SMS text messages during the intervention.

For peer review only - http://bmjopen.bmj.com/site/about/guidelines.xhtml

The non-receiving participants in the intervention group were tracked and cross-checked using individual participant codes from the server, and then approached by each health facility to confirm whether they were to be followed-up.

Changing phone numbers/SIM cards, phones being damaged, lost, or shared with others were identified as participant-related reasons for not receiving the messages, and were registered in the logbooks, and corrective measures were used either to replace phones with new devices or repair/maintain the phones as soon as was feasible.

Failing text message delivery influences medication intake

The adolescents identified several reasons for not receiving messages. In connection with the technical aspect, the adolescents highlighted problems such as not having a charged phone battery, or having lost their phone. They described that sometimes the text messages reminders arrived after their medication time. Another issue observed by both healthcare providers and family members was a missing SIM card. Furthermore, the continuity in the text messages reminders was requested by the adolescents to continue the benefits.

> *"… the receiving time varies; sometimes I receive it 10 minutes before medication time, which is 3p.m. local time in the evening, and sometimes it arrives after I take the drug." (Adolescent, aged 13)*

Limitations in phone ownership

Sometimes adolescents were not permitted to use the telephone by themselves, due to restrictions from their family, as they thought that the adolescents were too young to use the telephone. Instead, the family used the telephone, which resulted in text messages reminders never reaching the adolescent. The absence of SIM cards may be because adolescents did not receive a SIM

For peer review only - http://bmjopen.bmj.com/site/about/guidelines.xhtml

For peer review only

card, or because someone else in the family was using them. The family reported that they passed information from healthcare providers to the adolescents. Some of the families lacked information about the intervention and did not know what to do when the telephone arrived. The healthcare providers emphasised that some families denied the adolescents use of the telephone, which could cause problems, and text messages reminders were not received. Furthermore, the importance of informing families about the intervention was stressed, as several adolescents were restricted from using the telephone.

"…I think families should be communicated with regarding why their children are provided with mobile phones from the hospital, so as to avoid denial to access or adults using the phones for themselves." (Healthcare provider, aged 36)

Technical limitations

One major issue stressed by adolescents, families, and healthcare providers, was that the electric power system went off quite often in their communities, which caused problems with receiving text messages reminders if the battery in the telephone was not charged. The adolescents usually did not experience problems with delays in text messages reminders, but they had observed that the text messages reminders did not always arrive on time. The messages could come long before their scheduled medication time or sometimes very close to or after their scheduled medication time. Sometimes the text message reminder was absent for entire days.

"…The message helped me a lot to remember my medication time. However, the skips in message delivery dates and untimely arrivals should be corrected so that it is more helpful." (Adolescent, aged 18)

Maintenance:

After initial configuration, a single server can send text messages to thousands of participants across a large geographic area. In this study, a server sent text messages to 153 participants across

For peer review only - http://bmjopen.bmj.com/site/about/guidelines.xhtml

5 different geographic areas. The technical challenges experienced in delivering the intervention were related to navigating the messaging server and feedback responses from the participants. The non-receiving participants in the intervention group were tracked and cross-checked using a four-digit individual participant codes from the server and were then approached by each facility to confirm whether they were indeed in that group or lost. Despite technical barriers, and adverse conditions, and a belief in the future of text messages reminders emerged, which was also expressed in the qualitative interviews.

Seeing potential in SMS services

The benefits of SMS reminders were perceived as valuable; especially when adolescents missed appointments for medication refills and virus load tests. Before the intervention, there were difficulties getting in touch with the adolescents, because they lived in rural areas and did not have a telephone. The healthcare providers thought about how to incorporate text messages reminders into routine health care. A wish among the adolescents was expressed that more text messages reminders could be helpful in raising awareness about their condition. Both the adolescents and the healthcare providers raised a need to continue the text message reminders.

> *"I think this must continue. The mobile provision has played significant roles in adherence and retention, as many of the adolescents do not have phones…because of this provision, adolescents can be reached directly with health messages and receive important lessons just from their phones." (Adolescent, aged 19)*

Possibilities with text messages reminders

The healthcare provider described that possibilities to communicate with the adolescents had increased after the intervention, and the adolescents' attitudes towards healthcare facilities had become more positive. The adolescents found the improved digital communication to be an

For peer review only - http://bmjopen.bmj.com/site/about/guidelines.xhtml

advantage, because the healthcare provider no longer needed to call their family but could call them directly.

> *"Previously, healthcare providers from hospital reached me through neighbourhood phone numbers. She now uses this number to reach me directly through the phone provided by the hospital. This is a benefit for my privacy and for the information we discuss over the phone." (Adolescent, aged 18)*

DISCUSSION

Our process evaluation which was based on the RE-AIM framework (18) showed that intervention fidelity was high with only about 0.6% of the SMS messages not delivered as intended. Both providers and patients described the SMS reminders as being helpful. A few barriers were noted including glitches at telecommunication networks, electric power disruptions, issues with phone ownership at household levels. Our results highlight that SMS message reminders can be delivered with high coverage if proper processes are followed. The 4-digit short code messaging system was a key enabler in ensuring confidentiality and security of the messages. Further scale-up of such interventions will depend on addressing broader systems issues including phone ownership, fixing network interruptions and reliable power sources. However, a study conducted in Philippines showed that intervention fidelity was low (77.9%) in the message group. The identified reason for low intervention fidelity was poor reliability of local telecommunication networks, and frequency of messages received (28). The local context for cellular phone infrastructure and operational challenges, such as multiple users on a single cell phone, has an impact on text messaging interventions (29). It is important to assess the setting before using the SMS intervention as a strategy to improve adherence. There is emerging evidence that mobile phones can play an important role in healthcare delivery, especially in resource-limited settings (30). SMS text messaging is a particularly useful application that can be used to collect or share information and

For peer review only - http://bmjopen.bmj.com/site/about/guidelines.xhtml

to enhance communication between healthcare personnel and patients in a low-cost manner (31). Treatment support was highlighted as an important factor for adolescents to avoid poor adherence. Text messages could be a facilitator for adolescents to remain hopeful and avoid unwanted side effects given that previous research (32, 33) has shown that a lack of support systems was perceived to negatively affect adherence among young people. Our findings provide additional evidence in support of research to identify effective interventions to improve adherence and retention in care for adolescents with HIV and enhance their engagement in these services. Thus, findings of this process evaluation can guide further research towards interventions concerning readiness among healthcare facilities, because settings can impact implementation. Giving importance to the voice of adolescents living with HIV can offer hope to many more in the same situation. This study thereby has a potential clinical impact and contributes to UN Sustainability Goal (34).

There are both strengths and limitations identified in this process evaluation. First, the intervention has several strengths, and a key strength is the use of a 4-digit short code for text messages that enables rapid, high-volume outbound messaging, is easy to read and remember, and assists text recipients in identifying the sender based on their preferences for message delivery time, message content, and messaging language. The use of the unique code can contribute to maintaining security and confidentiality among participants.

Secondly, the messaging server is adaptable and user-friendly, as the researcher or local IT provider can add and remove new data fields to navigate dropped or missed individuals and update SMS text message content to avoid message-related fatigue.

In addition, the process evaluation also has several strengths that were identified. The first strength is the use of mixed methods, where different forms of data—both quantitative and qualitative methods—are combined to capture the multi-dimensionality of the intervention. The data

For peer review only - http://bmjopen.bmj.com/site/about/guidelines.xhtml

supplement one another and aid in obtaining a comprehensive picture of the study results, which can contribute to a deeper understanding of the participants' experiences.(35) If the participants are given the opportunity to highlight their own individual priorities, it might be possible for us to improve our understanding of the impact of the intervention.

Finally, three different perspectives (adolescents, families, and healthcare providers) were included in the qualitative interviews, which allowed different voices to be heard. However, limited numbers of included participants from adolescents' families and healthcare providers can limit the study's transferability.(36)

Conclusion

The mobile phone text messaging intervention to support ART adherence and retention in care was well-received by participants, and overall intervention fidelity was high. However, the intervention's feasibility was dependent on the reliability of local telecommunication networks, local electric power, and monitoring of adolescents' families, all of which had a significant impact on the intervention's usability, fidelity, and doses received. The participants experienced the text messaging function as helpful, therefore the development of different aspects, such as addressing technical problems, would be desirable to further improve impact. Further research needs to be directed toward readiness within the healthcare facilities to capture an increased understanding of barriers and facilitators in implementation.

Author affiliations

[1]Child and Family Health Unit, Department of Health Sciences, Faculty of Medicine, Lund University, Lund, Sweden.

For peer review only - http://bmjopen.bmj.com/site/about/guidelines.xhtml

[2] School of Public Health, College of Medicine and Health Sciences, Arba Minch University, Arba Minch, Ethiopia.

[3] KNCV Tuberculosis Foundation, The Hague, Netherlands.

Acknowledgements

The authors sincerely thank the participants for sharing their experiences, and all staff working in the ART clinics for facilitating the study.

Contributors

AT, DJ, and IKH conceptualised and developed the study design. AT collected the data and managed study implementation. DJ and IKH supervised data collection and study implementation. All four authors contributed to data analysis and interpretation. AT and HÅP drafted the manuscript. All authors contributed to critical revision of the manuscript and approved the final manuscript.

Funding

The project was externally funded by FORTE: Swedish Research Council for Health, Working Life and Welfare Life (FORTE) (https://forte.se/en/) program support 2018–01399 and the Swedish Research Council(https://www.vr.se/english.html) program support VR 2016-05706. The study protocol has undergone a peer review by the funding body. The funders had no role in study design, writing the study protocol, the decision to publish the study protocol, or preparation of the manuscript.

Competing interests

None declared.

For peer review only - http://bmjopen.bmj.com/site/about/guidelines.xhtml

Patient and public involvement

Patient consent for publication

Not applicable.

Ethics approval

The study was conducted according to the guidelines in the Declaration of Helsinki (37) and approved by the Swedish Regional Ethical Review Board (Dnr 2019-03433), National Research Ethics Review Committee in Ethiopia (MoSHE/RD/142/2869/20), and the Institutional Research Ethics Review Board in Arba Minch University (IRB-113/11). The participants received both oral and written information about the purpose of the study, about the confidential treatment of the data and the voluntary nature of participation. Informed consent was provided from the participants before the study began.

Provenance and peer review

Not commissioned, externally peer reviewed.

Data availability statement

No datasets were generated or analysed during the current study. All relevant data from this study will be made available upon reasonable written request and in accordance with ethical approval, from the corresponding author.

Supplemental material

Open access

For peer review only - http://bmjopen.bmj.com/site/about/guidelines.xhtml

This is an open access article distributed in accordance with the Creative Commons Attribution Non-Commercial (CC BY-NC 4.0) license, which permits others to distribute, remix, adapt, build upon this work non-commercially, and license their derivative works on different terms, provided the original work is properly cited, appropriate credit is given, any changes made indicated, and the use is non-commercial. See: http://creativecommons. org/licenses/ by-nc/ 4.0/

ORCID

Abayneh Tunje (orcid.org/0000-0002-0957-0216)
Degu Jerene (orcid.org/ 0000-0001-7853-4211)
Helene Åvik Persson (orcid.org/0000-0002-7862-5444)
Inger Kristensson Hallström (orcid.org/0000-0002-9402-6230)

References

1. UNAIDS. Global HIV & AIDS statistics — Fact sheet: https://www.unaids.org/en/resources/fact-sheet. 2021.
2. UNICEF. Global and regional trends. 2022 [Available from: https://data.unicef.org/topic/hivaids/global-regional-trends/.
3. Duncombe C, Ravishankar S, Zuniga JM. Fast-Track Cities: striving to end urban HIV epidemics by 2030. Current Opinion in HIV and AIDS. 2019;14(6):503-8.
4. World Health Organization. HIV data and statistics. World Health Organization (WHO) Available online: https://www who int/teams/global-hiv-hepatitis-and-stis-programmes/hiv/strategic-information/hiv-data-and-statistics. 2021.
5. African Network for the Care of Children Affected by HIV/AIDS – ANECCA. Handbook on Pædiatric AIDS in Africa by the African Network for the Care of Children Affected by AIDS 2017. Available

For peer review only - http://bmjopen.bmj.com/site/about/guidelines.xhtml

from: https://www.anecca.org/wp-content/uploads/2018/12/ANECCA-HANDBOOK-2017-ENGLISH-VERSION_20170530_web.pdf.
6. Pugh LE, Roberts JS, Viswasam N, Hahn E, Ryan S, Turpin G, et al. SYSTEMATIC REVIEW OF INTERVENTIONS AIMED AT IMPROVING HIV ADHERENCE TO CARE IN LOW-AND MIDDLE-INCOME COUNTRIES. Journal of Infection and Public Health. 2022.
7. Sefah IA, Mensah F, Kurdi A, Godman B. Barriers and facilitators of adherence to antiretroviral treatment at a public health facility in Ghana: a mixed method study. Hospital Practice. 2022;50(2):110-7.
8. Hudelson C, Cluver L. Factors associated with adherence to antiretroviral therapy among adolescents living with HIV/AIDS in low-and middle-income countries: a systematic review. AIDS care. 2015;27(7):805-16.
9. Rudy BJ, Murphy DA, Harris DR, Muenz L, Ellen J. Patient-related risks for nonadherence to antiretroviral therapy among HIV-infected youth in the United States: a study of prevalence and interactions. AIDS patient care and STDs. 2009;23(3):185-94.
10. Gill MM, Ndimbii JN, Otieno-Masaba R, Ouma M, Jabuto S, Ochanda B. Adherence challenges and opportunities for optimizing care through enhanced adherence counseling for adolescents with suspected HIV treatment failure in Kenya. BMC Health Services Research. 2022;22(1):1-11.
11. MacDonell K, Naar-King S, Huszti H, Belzer M. Barriers to medication adherence in behaviorally and perinatally infected youth living with HIV. AIDS and Behavior. 2013;17(1):86-93.
12. Ajuna N, Tumusiime B, Amanya J, Awori S, Rukundo GZ, Asiimwe JB. Social networks and barriers to ART adherence among young adults (18–24 years) living with HIV at selected primary health facilities of South-Western Uganda: A qualitative study. HIV/AIDS (Auckland, NZ). 2021;13:939.
13. Thompson TL, Haskard-Zolnierek K. Adherence and communication. Oxford Research Encyclopedia of Communication2016.
14. MacPherson P, Munthali C, Ferguson J, Armstrong A, Kranzer K, Ferrand RA, et al. Service delivery interventions to improve adolescents' linkage, retention and adherence to antiretroviral therapy and HIV care. Tropical medicine & international health. 2015;20(8):1015-32.
15. Bervell B, Al-Samarraie H. A comparative review of mobile health and electronic health utilization in sub-Saharan African countries. Social Science & Medicine. 2019;232:1-16.
16. Free C, Phillips G, Galli L, Watson L, Felix L, Edwards P, et al. The effectiveness of mobile-health technology-based health behaviour change or disease management interventions for health care consumers: a systematic review. PLoS medicine. 2013;10(1):e1001362.
17. Creswell JW. A concise introduction to mixed methods research: SAGE publications; 2014.
18. Glasgow RE, Harden SM, Gaglio B, Rabin B, Smith ML, Porter GC, et al. RE-AIM planning and evaluation framework: adapting to new science and practice with a 20-year review. Frontiers in public health. 2019;7:64.
19. Graneheim UH, Lindgren B-M, Lundman B. Methodological challenges in qualitative content analysis: A discussion paper. Nurse education today. 2017;56:29-34.
20. Abiodun O, Ladi-Akinyemi B, Olu-Abiodun O, Sotunsa J, Bamidele F, Adepoju A, et al. A single-blind, parallel design RCT to assess the effectiveness of SMS reminders in improving ART adherence among adolescents living with HIV (STARTA trial). Journal of Adolescent Health. 2021;68(4):728-36.
21. Pop-Eleches C, Thirumurthy H, Habyarimana JP, Zivin JG, Goldstein MP, De Walque D, et al. Mobile phone technologies improve adherence to antiretroviral treatment in a resource-limited setting: a randomized controlled trial of text message reminders. AIDS (London, England). 2011;25(6):825.
22. Craig P, Dieppe P, Macintyre S, Michie S, Nazareth I, Petticrew M. Developing and evaluating complex interventions: the new Medical Research Council guidance. Bmj. 2008;337.

For peer review only

For peer review only - http://bmjopen.bmj.com/site/about/guidelines.xhtml

23. Mehra N, Tunje A, Hallström IK, Jerene D. Effectiveness of mobile phone text message reminder interventions to improve adherence to antiretroviral therapy among adolescents living with HIV: A systematic review and meta-analysis. Plos one. 2021;16(7):e0254890.
24. Tunje A, Jerene D, Kristensson Hallström I. Antiretroviral Therapy and Retention in Care Experiences and Needs of Adolescents Living with HIV in Southern Ethiopia. HIV/AIDS-Research and Palliative Care. 2021:999-1007.
25. McPherson AC, Gofine ML, Stinson J. Seeing is believing? A mixed-methods study exploring the quality and perceived trustworthiness of online information about chronic conditions aimed at children and young people. Health Communication. 2014;29(5):473-82.
26. Spinuzzi C. The methodology of participatory design. Technical communication. 2005;52(2):163-74.
27. Lindgren B-M, Lundman B, Graneheim UH. Abstraction and interpretation during the qualitative content analysis process. International journal of nursing studies. 2020;108:103632.
28. O'Connor C, Leyritana K, Doyle AM, Birdthistle I, Lewis JJ, Gill R, et al. Delivering an mHealth adherence support intervention for patients with HIV: Mixed methods process evaluation of the Philippines connect for life study. JMIR Formative Research. 2022;6(8):e37163.
29. Sánchez SA, Ramay BM, Zook J, de Leon O, Peralta R, Juarez J, et al. Toward improved adherence: a text message intervention in an human immunodeficiency virus pediatric clinic in Guatemala City. Medicine. 2021;100(10).
30. Mukund Bahadur K-C, Murrayb PJ. Cell phone short messaging service (SMS) for HIV/AIDS in South Africa: a literature review. MEDINFO 2010. 2010:530-4.
31. Lester R, Karanja S. Mobile phones: exceptional tools for HIV/AIDS, health, and crisis management. The Lancet infectious diseases. 2008;8(12):738-9.
32. Hlophe LD, Tamuzi JL, Shumba C, Nyasulu PS. Barriers to anti-retroviral therapy adherence among adolescents aged 10 to 19 years living with HIV in sub-Saharan Africa: A mixed-methods systematic review protocol. Plos one. 2022;17(9):e0273435.
33. Ammon N, Mason S, Corkery J. Factors impacting antiretroviral therapy adherence among human immunodeficiency virus–positive adolescents in Sub-Saharan Africa: a systematic review. Public health. 2018;157:20-31.
34. United Nations Social and Economic Council. Progress towards the Sustainable Development Goals. Report of the Secretary-General. NY. USA; 2022.
35. Creswell JW, Klassen AC, Plano Clark VL, Smith KC. Best practices for mixed methods research in the health sciences. Bethesda (Maryland): National Institutes of Health. 2011;2013:541-5.
36. Lincoln YS, Guba EG. Naturalistic inquiry: sage; 1985.
37. World Medical Association. World Medical Association Declaration of Helsinki: ethical principles for medical research involving human subjects. Jama. 2013;310(20):2191-4.

For peer review only - http://bmjopen.bmj.com/site/about/guidelines.xhtml

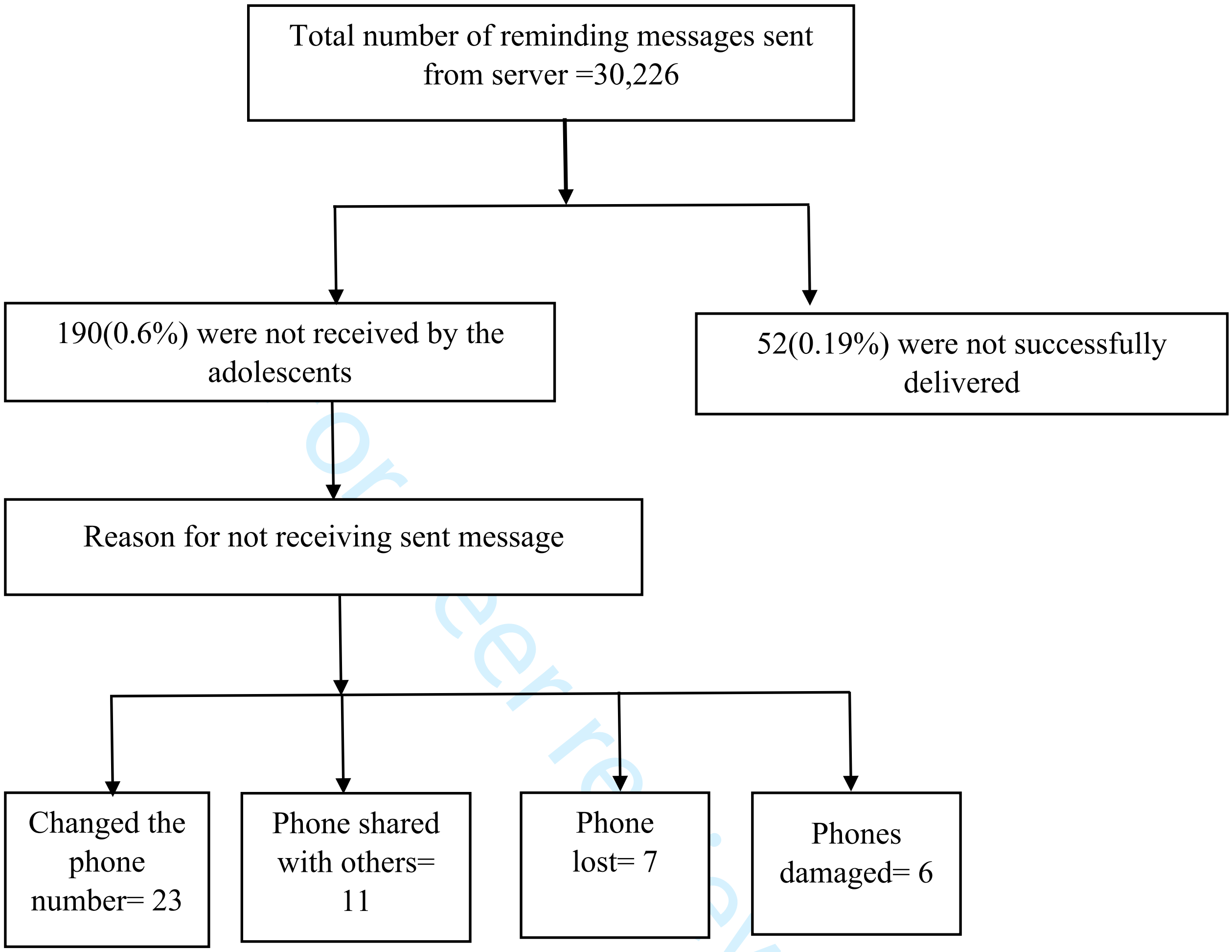

Figure 1. The flow diagram of mobile phone text messaging reminder in the intervention group

For peer review only

BMJ Open

Intervention fidelity and factors affecting the process of implementing a mobile phone text messaging intervention among adolescents living with HIV: a convergent mixed-methods study in southern Ethiopia

Journal:	*BMJ Open*
Manuscript ID	bmjopen-2023-077128.R1
Article Type:	Original research
Date Submitted by the Author:	07-Dec-2023
Complete List of Authors:	Tunje, Abayneh; Lund University Faculty of Medicine, Department of Health Sciences; Arba Minch University, Public health<br>Persson, Helene ; Lund University Faculty of Medicine, Department of Health Sciences<br>Jerene, Degu; KNCV Tuberculosis Foundation,<br>Hallstrom, Inger; Lunds Universitet, Department of Health Sciences
**Primary Subject Heading**:	HIV/AIDS
Secondary Subject Heading:	Infectious diseases, Public health
Keywords:	Adolescent, HIV & AIDS < INFECTIOUS DISEASES, Information technology < BIOTECHNOLOGY & BIOINFORMATICS

SCHOLARONE™
Manuscripts

I, the Submitting Author has the right to grant and does grant on behalf of all authors of the Work (as defined in the below author licence), an exclusive licence and/or a non-exclusive licence for contributions from authors who are: i) UK Crown employees; ii) where BMJ has agreed a CC-BY licence shall apply, and/or iii) in accordance with the terms applicable for US Federal Government officers or employees acting as part of their official duties; on a worldwide, perpetual, irrevocable, royalty-free basis to BMJ Publishing Group Ltd ("BMJ") its licensees and where the relevant Journal is co-owned by BMJ to the co-owners of the Journal, to publish the Work in this journal and any other BMJ products and to exploit all rights, as set out in our licence.

The Submitting Author accepts and understands that any supply made under these terms is made by BMJ to the Submitting Author unless you are acting as an employee on behalf of your employer or a postgraduate student of an affiliated institution which is paying any applicable article publishing charge ("APC") for Open Access articles. Where the Submitting Author wishes to make the Work available on an Open Access basis (and intends to pay the relevant APC), the terms of reuse of such Open Access shall be governed by a Creative Commons licence – details of these licences and which Creative Commons licence will apply to this Work are set out in our licence referred to above.

Other than as permitted in any relevant BMJ Author's Self Archiving Policies, I confirm this Work has not been accepted for publication elsewhere, is not being considered for publication elsewhere and does not duplicate material already published. I confirm all authors consent to publication of this Work and authorise the granting of this licence.

For peer review only

Intervention fidelity and factors affecting the process of implementing a mobile phone text messaging intervention among adolescents living with HIV: a convergent mixed methods study in southern Ethiopia

Abayneh Tunje, PhD-student[1,2*] abayneh_tunje.tanga@med.lu.se, Helene Åvik Persson, PHN, PhD[1] helene.avik_persson@med.lu.se, Degu Jerene, MD, PhD[3] degu.jerene.7501@med.lu.se, Inger Kristensson Hallström, RN, PhD, Professor[1] inger.kristensson_hallstrom@med.lu.se

Affiliations:

[1]Child and Family Health Unit, Department of Health Sciences, Faculty of Medicine, Lund University, Lund, Sweden.

[2] School of Public Health, College of Medicine and Health Sciences, Arba Minch University, Arba Minch, Ethiopia.

[3] KNCV Tuberculosis Foundation, The Hague, Netherlands.

***Corresponding author:**

Abayneh Tunje
abayneh_tunje.tanga@med.lu.se
The Faculty of Medicine
Department of Health Sciences
P.O. Box 157
221 00 Lund, Sweden

For peer review only

Abstract

Objective: To assess the intervention fidelity and explore contextual factors affecting the process of implementing a mobile phone text messaging intervention in improving adherence to and retention in care among adolescents living with HIV, their families, and their healthcare providers in southern Ethiopia.

Design: A convergent mixed-methods design guided by the process evaluation theoretical framework and the RE-AIM framework was used alongside a randomised controlled trial to examine the fidelity and explore the experiences of participants in the intervention.

Setting: Six hospitals and five health centres providing HIV treatment and care to adolescents in five zones in southern Ethiopia.

Participants: Adolescents (aged 10–19), their families and their healthcare providers.

Intervention: Mobile phone text messages daily for 6 months or standard care (control).

Results: 306 participants were enrolled in the process evaluation. Among the intervention participants (N =153), 171 (55.9%) of whom were men, most resided in an urban area 225 (73.5%), and participants had a mean age of 15 (2.62). The overall experiences of implementing the text messages reminder intervention were described as helpful in terms of treatment support for adherence but had room for improvement. During the study, 30,700 text messages were sent, and fidelity was high, with 99.4% successfully receiving text messages during the intervention. Barriers such as failed text messages delivery, limitations in phone ownership, and technical limitations affected fidelity. Technical challenges can hinder maintenance, but a belief in the future of digital communication permeates the experiences of the text messages reminders.

For peer review only - http://bmjopen.bmj.com/site/about/guidelines.xhtml

Conclusions: Overall fidelity was high, and participants' overall experiences of mobile phone text messages were expressed as helpful. Contextual factors, such as local telecommunications networks and local electric power, as well as technical and individual factors must be considered when planning future interventions.

Trial registration: Pan African Clinical Trials **PACTR202107638293593, first registration** 20/07/2021.

Strengths and limitations with this study

- Secure and confidential delivery of high-volume messages through a 4-digit short code.
- The use of a mixed methods design and the inclusion of family members and healthcare providers is a strength of this process evaluation study.
- There was a limited number of adolescents´ family and healthcare providers included in the interviews.

For peer review only

INTRODUCTION

Globally, 38.4 million people were living with HIV in 2021. Of these, 1.71 million were adolescents aged 10–19, 88% of whom live in Sub-Saharan Africa (1, 2). To control the epidemic, the Joint United Nations Programme on HIV/AIDS (UNAIDS) launched the 95%–95%–95% targets aimed at HIV testing, treatment, and viral suppression by 2030 (3). However, by 2021, 75% percent of the 38.4 million people living with HIV (PLHIV) were receiving antiretroviral therapy (ART), a treatment gap of 5.9 million people (4). Moreover, achieving the recommended optimal adherence level of >95% which is required for ART to be effective, remains a challenge within the HIV care continuum (5). This poses a significant threat to HIV interventions in resource-limited settings (6).

Poor adherence to ART has several consequences, including increased risk of viral drug resistance and reduced treatment effectiveness toward viral suppression, leading to disease progression, greater risk of death, and increased risk of viral transmission (7). Some of the major barriers to ART adherence include socioeconomic status,(8, 9) fear of being stigmatised or discriminated against as a result of one's HIV status,(10) forgetting to take medication on time,(11) treatment fatigue,(12) and patient–provider communication (13). Several interventions to overcome these barriers have been studied in a variety of settings (14). The use of digital health strategies to enhance HIV treatment is one of the priority interventions in Sub-Saharan Africa (15). Targeted digital client communication using mobile phone-based short text messaging has been recommended for adherence support for a range of health issues and adherence to antiretroviral treatment (16). The success and effectiveness of an mHealth intervention in achieving desired health outcomes is dependent on the context in which they are implemented (15, 16). Context plays a crucial role in either the success or failure of an intervention.

For peer review only - http://bmjopen.bmj.com/site/about/guidelines.xhtml

In addition to design and implementation, the success of interventions is diverse and may be influenced by factors such as sociodemographic and culture (17). Observing what is delivered in practice with close reference to the theory of the intervention can help evaluators in distinguishing the intervention fit in different contexts and changes that undermine intervention fidelity (15, 17). Trials should continue to rigorously assess outcomes, but also include integral process evaluations that use qualitative and quantitative data to develop and test hypotheses about how interventions work (18). In a multi-centre trial, a process evaluation is necessary to understand whether the intervention was implemented and received similarly across all sites (9). However, trials assessing mHealth adherence interventions for HIV often do not include process evaluations to examine the fidelity and quality of the intervention delivery, causal mechanisms for the health outcomes, contextual factors affecting the delivery, and costs of implementation (16, 19). Understanding the entire implementation process of an mHealth intervention allows practitioners to interpret the results and replicate the intervention in other contexts (20, 21). Therefore, this study aimed to evaluate the process of a mobile text messaging intervention conducted among adolescents living with HIV in Ethiopia.

For peer review only

For peer review only - http://bmjopen.bmj.com/site/about/guidelines.xhtml

METHODS

Study design

A convergent mixed-methods design (17) combining quantitative and qualitative techniques was used. The design was guided by the process evaluation theoretical framework (22) and structured by the RE-AIM dimensions (Reach, Effectiveness, Adoption, Implementation and Maintenance) (18) to evaluate intervention effectiveness alongside a randomised controlled trial (RCT) to examine the study's fidelity and to explore the experiences of the participants in the intervention.

The trial was registered in Pan African Clinical Trials Registry with the registration number PACTR202107638293593 on 17/07/2021.

Study setting and participants

Six hospitals and five health centres that provide HIV treatment and care to adolescents in the Gamo, Gofa, Konso, South Omo, and Wolayita zones in Southern Ethiopia were included in the main study. These zones are home to over 25 distinct ethnic groups, each with its own culture and context. Between July 5, 2022, and February 28, 2023, 306 adolescents living with HIV were included in the study, in either the intervention or in the control group. The quantitative data included process indicators, while the qualitative interviews included ten adolescents from the main trial, four adolescents' families, and two healthcare providers who were involved in the process evaluation. We used the following technique to identify eligible adolescents: first, we identified the number of adolescents of eligible age in each facility. Then, using their address, we called each adolescent and their parents or caregivers directly via phone numbers, care providers, or adherence supports. Then, data collectors approached adolescents and posed literacy questions about their ability to read text by displaying sample texts. Then, eligible participants in the study were asked to give consent and assent. Thus, adolescents aged 10 to 19 years, diagnosed with HIV, currently

For peer review only - http://bmjopen.bmj.com/site/about/guidelines.xhtml

participating in ART care, and planning to stay in the designated facility for at least six months following study enrolment were eligible. Participants who were above the age limit, did not disclose their HIV status, were unable to read text, or had a disability were all excluded. Finally, the data collector showed each eligible adolescent how to use their mobile device, including text saving and deletion.

Theoretical Framework of Process Evaluation

The RE-AIM framework (18) was used for the process evaluation to comprehensively examine the level of implementation. This involves understanding the implementation of interventions in terms of (1) fidelity, defined as to what extent the intervention was implemented consistently with the underlying theory as planned; (2) delivery, defined as to what extent all of the intended activities, training, and materials were provided to program participants; (3) reach, defined as the absolute number, proportion, and representativeness of individuals who are willing to participate in a given initiative, intervention, or program; (4) receipt, defined as how participants reacted to specific aspects of the intervention; and (5) context, defined as what contextual factors influence implementation or the intervention outcome.

Description of the intervention

The MRC framework,(22) a systematic literature review,(23) interviews with adolescents,(24) and the theoretical health belief model (HBM) (25) were used to develop the intervention. Adolescents living with HIV, healthcare providers, and research project technical advisory team members participated in the design (26) under the guidance of a research advisory committee. The HBM is a theoretical model that has been constructed from six domains: susceptibility, severity, barriers, benefit, cues to action, and self-efficacy. After the intervention's development, text messages were uploaded to the server, and an automated message-pushing software was used to send tailored daily

For peer review only - http://bmjopen.bmj.com/site/about/guidelines.xhtml

text messages using a four-digit code from Ethiopia Telecom. This code helps adolescents in avoiding messaging source-related stigma and distinguishing intervention messages from other messages.

Other various configurations such as wide-area network (WAN) internet protocol (IP), local area network (LAN) IP, Ethio telecom central server IP, a virtual private network (VPN) line, and office servers were used as infrastructure, as required to support the study. The text messages were categorised and delivered based on each adolescent's customised medication schedule and time. The software sent the message 15 minutes before each participant's medication time. The type of message was short and mainly focused on advice about the benefits of taking on time, the risk/consequences of missing/not taking on time, encouragement, and without mentioning anything about HIV or the name of the drug. For example, "Those who use it properly know its benefits", "I'm taking it properly and on time". For example, these messages in Amharic are "በትክክል የሚጠቀሙት ጥቅሞቹን ያውቃሉ) and በትክክል እና በሰዓት እወስዳለሁ respectively. The server was checked daily to troubleshoot network/cell phone issues. Based on the server information, the first author contacted the facility level care provider to contact the adolescent with corrective actions; for example, subscriber identity module card (SIM card) replacement, damaged mobile phones, and network troubleshooting for maintenance and replacement.

For peer review only

For peer review only - http://bmjopen.bmj.com/site/about/guidelines.xhtml

Sample and data collection

The quantitative data was collected from the server, activity logs, an adverse event monitoring form, and an interview with adolescents. The researcher collects data on intervention fidelity by tracking the number of text messages sent, received, and failed from the server daily. Furthermore, each facility representative documented any adverse event related to the trial, filled the form, and sent it to researchers every week. Furthermore, during the follow-up data collection phase, adolescents asked if they received text messages on a regular schedule.

A purposive sampling of 10 adolescents with HIV (aged 12–19), four family members/relatives (aged 36–45), and two healthcare providers (aged 31–36) from five different healthcare facilities were asked to be interviewed about their experiences in the intervention between June and July 2022 at healthcare facilities by the first author. The interviews were conducted in all respondent languages, such as Amharic, Wolaytigna, Gamogna, Gofigna, Arigna, and Konsegna in a quiet and confidential area at the respective health facilities by the first author and one research assistant and lasted on average 30 minutes.

An interview guide was used to gain a rich, in-depth understanding, and to allow the participants to concretise their experiences of their use of text messages, communication, and technical aspects of the intervention. The interview guide included subsections adapted to the participants, i.e., adolescents, families, and healthcare providers. Hence, the questions were different, depending on to whom they were addressed. The introductory question to the adolescents was: "Are you receiving the mobile text-message reminder from the hospital?", followed by follow-up questions such as, "What do you think about the messages?" The families were initially asked "How do family/social related factors affect ART adherence among adolescents?" Healthcare providers were

For peer review only - http://bmjopen.bmj.com/site/about/guidelines.xhtml

asked "How did you experience communication with the adolescents with regard to the intervention?" Follow-up questions and prompts were used throughout the whole interview.

The interviews were audiotaped, transcribed verbatim, and translated from Amharic to English by the first author, and checked for accuracy by one independent language expert.

Data analysis

The number of SMS messages sent and delivered to participants were tracked as a measure of intervention fidelity. When SMS messages did not arrive on the phone, the project advisory team investigated the external network and other infrastructure issues. If the message could not be delivered during this period, the computer automatically recorded it as a delivery failure and sent the message again. Data captured on the delivery status of the SMS messages were recorded as: delivered (the phone had reception marked as green); undelivered (the phone was switched off or marked as red in the server). The program could not record whether messages delivered to the phones were opened. The descriptive statistical analysis was used to summarize data in frequency, percentages, mean and standard deviation. The quantitative effectiveness data is analyzed separately and not included in the process evaluation. The RE-AIM outcomes framework components of Reach, Effectiveness, Adoption, Implementation, and Maintenance were used to present mixed method findings about the delivery of a reminder message, success, and intervention barriers for the intervention implementation process (18).

Interviews were analysed by using the principles for content analysis described by Graneheim and Lundman (19, 27). First, the transcripts were read several times to gain a sense of the whole by the first and the last author. Thereafter, meaning units were identified and condensed to reduce the text while maintaining the core meaning. The first author developed a coding frame for the analysis by using NVivo software, which were discussed by all authors. Condensed meaning units were then

For peer review only - http://bmjopen.bmj.com/site/about/guidelines.xhtml

labelled with a code, which were kept close to the text on a manifest level. Next, sub-themes were created from the code frame by the first and last authors. The sub-themes were then abstracted into themes. Finally, the underlying meaning—the latent content—was described into a main theme. Thereafter one of the authors (HÅP) read the transcripts, extracted meaning units, codes, and themes. The content was discussed and reflected upon together with all authors and lasted until an agreement was reached.

Patient and Public Involvement

Representatives of adolescents were involved in the development of messages for the SMS reminders and interview guides. Their feedback was used to adapt its contents and to set strategies to promote inclusivity in study participation during participant recruitment. In addition, participants, healthcare providers, and the participants' families were involved in reporting any incidence related to mobile devices and other issues.

For peer review only - http://bmjopen.bmj.com/site/about/guidelines.xhtml

249 **RESULTS**

250 **The characteristics of adolescents assigned in the intervention arm are shown in table 1.**

251 Table 1. Participant characteristics

**Characteristics of adolescents assigned in the intervention**		
**Characteristics**		Frequency /Mean (SD)/ Percent
Adolescent age, Mean (standard deviation (SD))		15(0.21) years
Adolescents age category	10-14	60 (39.22)
	15-19	93 (60.78)
Adolescents gender	Male	78 (49.02)
	Female	75 (43.8)
Languages of adolescent during interview	Amharic	62 (40.52)
	Wolaytigna	27 (17.65)
	Gamogna	39 (25.49)
	Gofigna	13 (8.5)
	Arigna	6 (3.3)
	Konsegna	6 (3.3)
Educational status of adolescent	Grade 1-8	97 (63.40)
	Grade 9 and above	56 (36.60)
Residence of adolescent	Urban	111(72.55)
	rural	42 (27.45)
Living arrangement	Live alone	10 (6.54)
	Live with family	143 (93.46)
Psycho-social peer-support	Participate	47 (30.72)
	Do not Participate	106 (69.28)
Interviewee characteristics		
Adolescents (Sex/age)	Male (10-14 years)	2
	Female (10-14 years)	3
Adolescents (Sex/age)	Male (15-19 years)	2
	Female (15-19 years)	3
Adolescents level of education	Grade 1-8	5
	Grade 9 and above	5
healthcare providers (Sex/Age)	Male	1 (36 years)
	Female	1 (31 years)
Parent/care giver (Sex/Age)	Non-biological care giver	2 (1 male and 1 female, 36 and 45 years respectively
	Biological	2 (1 mother and 1 father, 39 and 45 years respectively)

For peer review only - http://bmjopen.bmj.com/site/about/guidelines.xhtml

The results are structured using the RE-AIM framework (18) and corresponding themes (Table 2).

Table 2. Overview of the qualitative themes related to the dimensions in the RE-AIM framework.

**RE-AIM dimension (18)**	**Definition (18)**	**Qualitative theme**
Reach	The number, proportion, and representativeness of eligible individuals who participate in each initiative.	
Effectiveness	The impact of an intervention on the relevant outcomes, including potential adverse effects, quality of life, and economic outcomes.	Text message reminders were helpful for adherence but there was room for improvement
		Text messages reminders support adherence
		Feeling comfortable receiving text messages reminders
		The code delivered with text messages reminders minimises the risk of stigma.
Adoption	The reach and effectiveness/efficacy of an intervention at the setting level.	Supporting the text messages reminders
Implementation	The intervention agents' fidelity to the various elements of an intervention's protocol, including consistency of intended delivery.	Failing text messages delivery influences medication intake
		Limitations in phone ownership
		Technical limitations
Maintenance	Connects with both setting-level indicators (the extent to which a program or a policy becomes part of organisational practices and policies), and individual-level indicators (monitoring of effectiveness of an intervention or program six months or more after the most recent contact).	Seeing potential in SMS services
		Possibilities of text messages reminders

For peer review only

Reach:

The total number of individuals that were assessed for eligibility was 435, of which 306 (70.34 %) meet eligibility criteria. The remaining 129 participants excluded for the following reasons: 27 (20.9%) were unable to stay in the study area for the duration of the study, 39 (30.2%) were unable to read, 24 (18.6%) were did not know their status, 21 (16.3%) were could not reached, 13 (10.1%) refused to participate, and 5 (3.9%) had an impairment. Thus, 306 participants were enrolled and randomised: n =153 in the intervention, and n =153 in the control group. Among the 153 enrolled in intervention arm, 78 (49.02%) were male, and 75 (43.8%) were female, respectively. The mean and standard deviation age of the participants is 15 (0.21). Of these, 111 (72.55%) resided in an urban area, 97 (63.40%) had primary school level education, and 10(6.54%) live alone(orphan).

Effectiveness:

The number of participants retained in the 6-month follow-up of the intervention group was higher than the control group in this study.

Text messages reminders were helpful for adherence but there was room for improvement

The overall experiences of the intervention among adolescents living with HIV was that text messages reminders were helpful when it came to their adherence to medication, but direct access to phones and better timing of messages should be assured to increase their functioning and usability. The retention rate was improved, and was the best achieved since the intervention was introduced. Despite this, more effort was requested concerning technical issues to make the reminders even more helpful. The participants described facilitating aspects like increased medication intake and communication. Also, the well-being of the adolescents and the support

For peer review only - http://bmjopen.bmj.com/site/about/guidelines.xhtml

function was highlighted. However, technical problems like lost telephones or text messages reminders arriving after medication time were emphasised as barriers.

Text messages reminders support adherence

They described that before the intervention, their medication was often forgotten, which resulted in it being taken later than it should have been due to forgetfulness. Sometimes the adolescent realised that they had forgotten to take their medication but then waited until dinner to take it. The adolescents described that text messages reminders were associated with taking their medicine and delays were avoided. Also, family members described situations when the adolescent's medication routines were interrupted, and where text messages reminders played a big role.

> *"…Yes, it supports treatment adherence, because most of time we follow the television program but sometimes the power goes off, we missed the exact time to see medication time. Currently this text message comes 10 to 15 minutes before our medication time." (Father, aged 45)*

Feeling positive about the text messages reminder

The text messages reminders contributed to positive attitudes and to decreased stigmatisation for adolescents, even when HIV/AIDS stigma is deeply rooted in the culture. Thus, the perception of the adolescents was that the text messages reminders played an important role for their overall well-being, because sometimes they had low moods. The text messages reminders were described as offering hope and supported the adolescents in achieving consistency in their medication adherence which helped them avoid, for example, fatigue.

> *"…The benefit of this message is it comes always and reminds me to take the drug on time. The other benefit is when I receive the message, I feel good." (Adolescent, aged 13)*

For peer review only - http://bmjopen.bmj.com/site/about/guidelines.xhtml

The code-delivered text messages reminders minimise the risk of stigma

The adolescent felt safe regarding privacy in the text messages reminders due to a code sent out to their telephone. The healthcare provider noticed a change in the views of both adolescents and family regarding increased trust and improved engagement with healthcare facilities during the intervention. Family expressed receiving coded text messages reminders could enable adolescents to live like other HIV-free adolescents. However, the adolescents indicated that after their family's HIV status was made public, they were subjected to stigma, which was reflected in the interview. Some members of the families were open with their HIV disclosure while others were not.

"My status has already been made public, and everybody knows about my family as well ... However, my husband lives in another area because of work, and knows his HIV status and makes things secret. He did not even take his medication when he come back home." (Mother, aged 45)

Adoption:

In this study, eleven health facilities with 153 participants received an intervention across 5 different geographic areas. According to healthcare providers, the rate of retention among adolescents has increased since the study began in the hospital, as described in the qualitative findings under a sub-theme.

".... By the way, in terms of retention, we have observed the best retention rate ever in this year since ART started in this hospital. Of course, our follow-up is there and there are other partners like CDC and ICAP. But this messaging has augmented the service we provide, and it increased our communication with them and helped them remind their medication irrespective of inconsistencies in messaging and medication time; the message delivery reminds them something targeted." (healthcare provider, aged 36)

For peer review only - http://bmjopen.bmj.com/site/about/guidelines.xhtml

Supporting the text messages reminders

The healthcare providers emphasised that text messages reminders were an important support for adolescents in their medication intake to avoid poor adherence. In addition to this, families indicated that they had a mission to support the adolescents in avoiding ART treatment fatigue. Several adolescents expressed that they had received support from their family concerning medication intake during the intervention. Thus, the text messages reminders were perceived as helpful, as they made participants feel happy and hopeful, even if they felt angered or were in a depressed mood that day.

> *"…The messages may help people pass good days, despite days that do not carry good fortune. It therefore helps people have good days and reminds them of medication times. One day, for example, being consumed with some ideas, I did not recall that I have medication to take; it is this text message that triggered me take my medicine."*
> *(Adolescent, aged 19)*

Implementation:

There were 306 mobile phones provided to adolescents during assignments into intervention and control groups. The advisory committee was involved in the implementation of the intervention using standard operational procedure (SOP). During the study, 30,700 SMS text messages were sent to 153 study participants in the intervention group (Figure 1).

Overall, intervention fidelity was high with (30,510/30,700) or 99.38% successfully received SMS text messages during the intervention.

The non-receiving participants in the intervention group were tracked and cross-checked using individual participant codes from the server, and then approached by each health facility to confirm whether they were to be followed-up.

For peer review only - http://bmjopen.bmj.com/site/about/guidelines.xhtml

Changing phone numbers/SIM cards, phones being damaged, lost, or shared with others were identified as participant-related reasons for not receiving the messages, and were registered in the logbooks, and corrective measures were used either to replace phones with new devices or repair/maintain the phones as soon as was feasible.

Failing text message delivery influences medication intake

The adolescents identified several reasons for not receiving messages. In connection with the technical aspect, the adolescents highlighted problems such as not having a charged phone battery or having lost their phone. During the feasibility study period, there was fluctuation in messaging time, and the text message reminders arrived after their medication time. Another issue observed by both healthcare providers and family members was a missing SIM card. Furthermore, the continuity in the text messages reminders was requested by the adolescents to continue the benefits.

> *"… the receiving time varies; sometimes I receive it 10 minutes before medication time, which is 3p.m. local time in the evening, and sometimes it arrives after I take the drug." (Adolescent, aged 13)*

Limitations in phone ownership

Sometimes adolescents were not permitted to use the telephone by themselves, due to restrictions from their family, as they thought that the adolescents were too young to use the telephone. Instead, the family used the telephone, which resulted in text messages reminders never reaching the adolescent. The absence of SIM cards may be because adolescents did not receive a SIM card, or because someone else in the family was using them. The family reported that they passed information from healthcare providers to the adolescents. Some of the families lacked information about the intervention and did not know what to do when the telephone arrived. The healthcare providers emphasised that some families denied the adolescents use of the telephone,

For peer review only - http://bmjopen.bmj.com/site/about/guidelines.xhtml

which could cause problems, and text messages reminders were not received. Furthermore, the importance of informing families about the intervention was stressed, as several adolescents were restricted from using the telephone.

"…I think families should be communicated with regarding why their children are provided with mobile phones from the hospital, so as to avoid denial to access or adults using the phones for themselves." (Healthcare provider, aged 36)

Technical limitations

One major issue stressed by adolescents, families, and healthcare providers, was that the electric power system went off quite often in their communities, which caused problems with receiving text messages reminders if the battery in the telephone was not charged. The adolescents usually did not experience problems with delays in text messages reminders, but they had observed that the text messages reminders did not always arrive on time. The messages could come long before their scheduled medication time or sometimes very close to or after their scheduled medication time. Sometimes the text message reminder was absent for entire days.

"…The message helped me a lot to remember my medication time. However, the skips in message delivery dates and untimely arrivals should be corrected so that it is more helpful." (Adolescent, aged 18)

Maintenance:

After initial configuration, a single server can send text messages to thousands of participants across a large geographic area. In this study, a server sent text messages to 153 participants across five different geographic areas. The technical challenges experienced in delivering the intervention were related to navigating the messaging server and feedback responses from the participants. The non-receiving participants in the intervention group were tracked and cross-checked using a four-digit individual participant codes from the server and were then approached by each facility to

For peer review only - http://bmjopen.bmj.com/site/about/guidelines.xhtml

confirm whether they were indeed in that group or lost. Despite technical barriers, and adverse conditions, and a belief in the future of text messages reminders emerged, which was also expressed in the qualitative interviews.

Seeing potential in SMS services

The benefits of SMS reminders were perceived as valuable; especially when adolescents missed appointments for medication refills and virus load tests. Before the intervention, there were difficulties getting in touch with the adolescents, because they lived in rural areas and did not have a telephone. The healthcare providers thought about how to incorporate text messages reminders into routine health care. A wish among the adolescents was expressed that more text messages reminders could be helpful in raising awareness about their condition. Both the adolescents and the healthcare providers raised a need to continue the text message reminders.

> *"I think this must continue. The mobile provision has played significant roles in adherence and retention, as many of the adolescents do not have phones…because of this provision, adolescents can be reached directly with health messages and receive important lessons just from their phones." (Adolescent, aged 19)*

Possibilities with text messages reminders

The healthcare provider described that possibilities to communicate with the adolescents had increased after the intervention, and the adolescents' attitudes towards healthcare facilities had become more positive. The adolescents found the improved digital communication to be an advantage because the healthcare provider no longer needed to call their family but could call them directly.

> *"Previously, healthcare providers from hospital reached me through neighbourhood phone numbers. She now uses this number to reach me directly through the phone*

For peer review only - http://bmjopen.bmj.com/site/about/guidelines.xhtml

provided by the hospital. This is a benefit for my privacy and for the information we discuss over the phone." (Adolescent, aged 18)

DISCUSSION

Our process evaluation which was based on the RE-AIM framework (18) showed that intervention fidelity was high with only about 0.6% of the SMS messages not delivered as intended. Both providers and patients described the SMS reminders as being helpful. A few barriers were noted including glitches at telecommunication networks, electric power disruptions, issues with phone ownership at household levels. Our results highlight that SMS message reminders can be delivered with high coverage if proper processes are followed. The 4-digit short code messaging system was a key enabler in ensuring confidentiality and security of the messages. Further scale-up of such interventions will depend on addressing broader systems issues including phone ownership, fixing network interruptions and reliable power sources. However, a study conducted in Philippines showed that intervention fidelity was low (77.9%) in the message group. The identified reason for low intervention fidelity was poor reliability of local telecommunication networks, and frequency of messages received (28). The local context for cellular phone infrastructure and operational challenges, such as multiple users on a single cell phone, has an impact on text messaging interventions (29). It is important to assess the setting before using the SMS intervention as a strategy to improve adherence. There is emerging evidence that mobile phones can play an important role in healthcare delivery, especially in resource-limited settings (30). SMS text messaging is a particularly useful application that can be used to collect or share information and to enhance communication between healthcare personnel and patients in a low-cost manner (31). Treatment support was highlighted as an important factor for adolescents to avoid poor adherence. Text messages could be a facilitator for adolescents to remain hopeful and avoid unwanted side effects given that previous research (32, 33) has shown that a lack of support systems was perceived

For peer review only - http://bmjopen.bmj.com/site/about/guidelines.xhtml

to negatively affect adherence among young people. Our findings provide additional evidence in support of research to identify effective interventions to improve adherence and retention in care for adolescents with HIV and enhance their engagement in these services. Thus, findings of this process evaluation can guide further research towards interventions concerning readiness among healthcare facilities, because settings can impact implementation. Giving importance to the voice of adolescents living with HIV can offer hope to many more in the same situation. This study thereby has a potential clinical impact and contributes to UN Sustainability Goal (34).

Methodological concerns/ limitations

There are both strengths and limitations identified in this process evaluation. First, the intervention has several strengths, and a key strength is the use of a 4-digit short code for text messages that enables rapid, high-volume outbound messaging, is easy to read and remember, and assists text recipients in identifying the sender based on their preferences for message delivery time, message content, and messaging language. The use of the unique code can contribute to maintaining security and confidentiality among participants.

Secondly, the messaging server is adaptable and user-friendly, as the researcher or local IT provider can add and remove new data fields to navigate dropped or missed individuals and update SMS text message content to avoid message-related fatigue.

In addition, the process evaluation also has several strengths that were identified. The first strength is the use of mixed methods, where different forms of data—both quantitative and qualitative methods—are combined to capture the multi-dimensionality of the intervention. The data supplement one another and aid in obtaining a comprehensive picture of the study results, which can contribute to a deeper understanding of the participants' experiences (35). If the participants

For peer review only - http://bmjopen.bmj.com/site/about/guidelines.xhtml

are given the opportunity to highlight their own individual priorities, it might be possible for us to improve our understanding of the impact of the intervention.

Finally, three different perspectives (adolescents, families, and healthcare providers) were included in the qualitative interviews, which allowed different voices to be heard. However, limited numbers of included participants from adolescents' families and healthcare providers can limit the study's transferability (36).

Conclusion

The mobile phone text messaging intervention to support ART adherence and retention in care was well-received by participants, and overall intervention fidelity was high. However, the intervention's feasibility was dependent on the reliability of local telecommunication networks, local electric power, and monitoring of adolescents' families, all of which had a significant impact on the intervention's usability, fidelity, and doses received. The participants experienced the text messaging function as helpful, therefore the development of different aspects, such as addressing technical problems, would be desirable to further improve impact. Further research needs to be directed toward readiness within the healthcare facilities to capture an increased understanding of barriers and facilitators in implementation.

Author affiliations

[1]Child and Family Health Unit, Department of Health Sciences, Faculty of Medicine, Lund University, Lund, Sweden.

[2] School of Public Health, College of Medicine and Health Sciences, Arba Minch University, Arba Minch, Ethiopia.

[3] KNCV Tuberculosis Foundation, The Hague, Netherlands.

For peer review only - http://bmjopen.bmj.com/site/about/guidelines.xhtml

Acknowledgements

The authors sincerely thank the participants for sharing their experiences, and all staff working in the ART clinics for facilitating the study.

Contributors

AT, DJ, and IKH conceptualized and developed the study design. AT collected the data and transcribed verbatim and translated from Amharic to English. DJ and IKH supervised data collection and study implementation. All authors read the transcripts, extracted meaning units, codes, and data analysis. Sub-themes were created from the code frame by the first and last authors. AT and HÅP drafted the manuscript, and all authors contributed to critical revision and approved the final manuscript.

Funding

The project was externally funded by FORTE: Swedish Research Council for Health, Working Life and Welfare Life (FORTE) (https://forte.se/en/) program support 2018–01399 and the Swedish Research Council(https://www.vr.se/english.html) program support VR 2016-05706. The study protocol has undergone a peer review by the funding body. The funders had no role in study design, writing the study protocol, the decision to publish the study protocol, or preparation of the manuscript.

Competing interests

None declared.

Patient and public involvement

Patient consent for publication

For peer review only - http://bmjopen.bmj.com/site/about/guidelines.xhtml

Not applicable.

Ethics approval

The study was conducted according to the guidelines in the Declaration of Helsinki (37) and approved by the Swedish Regional Ethical Review Board (Dnr 2019-03433), National Research Ethics Review Committee in Ethiopia (MoSHE/RD/142/2869/20), and the Institutional Research Ethics Review Board in Arba Minch University (IRB-113/11). The participants received both oral and written information about the purpose of the study, about the confidential treatment of the data and the voluntary nature of participation. Informed consent was provided from all participants over the age of 18 before the study began. Similarly, parental consent and adolescent assent were obtained from all participants under the age of 18 before the start of the trial.

For peer review only

Provenance and peer review

Not commissioned, externally peer reviewed.

Data availability statement

No datasets were generated or analysed during the current study. All relevant data from this study will be made available upon reasonable written request and in accordance with ethical approval, from the corresponding author.

Supplemental material

Open access

This is an open access article distributed in accordance with the Creative Commons Attribution Non-Commercial (CC BY-NC 4.0) license, which permits others to distribute, remix, adapt, build

For peer review only - http://bmjopen.bmj.com/site/about/guidelines.xhtml

upon this work non-commercially, and license their derivative works on different terms, provided the original work is properly cited, appropriate credit is given, any changes made indicated, and the use is non-commercial. See: http://creativecommons. org/licenses/ by-nc/ 4.0/

ORCID

Abayneh Tunje (orcid.org/0000-0002-0957-0216)
Degu Jerene (orcid.org/ 0000-0001-7853-4211)
Helene Åvik Persson (orcid.org/0000-0002-7862-5444)
Inger Kristensson Hallström (orcid.org/0000-0002-9402-6230)

For peer review only - http://bmjopen.bmj.com/site/about/guidelines.xhtml

References

1. UNAIDS. Global HIV & AIDS statistics — Fact sheet: https://www.unaids.org/en/resources/fact-sheet. 2021.
2. UNICEF. Global and regional trends. 2022 [Available from: https://data.unicef.org/topic/hivaids/global-regional-trends/.
3. Duncombe C, Ravishankar S, Zuniga JM. Fast-Track Cities: striving to end urban HIV epidemics by 2030. Current Opinion in HIV and AIDS. 2019;14(6):503-8.
4. World Health Organization. HIV data and statistics. World Health Organization (WHO) Available online: https://www who int/teams/global-hiv-hepatitis-and-stis-programmes/hiv/strategic-information/hiv-data-and-statistics. 2021.
5. African Network for the Care of Children Affected by HIV/AIDS – ANECCA. Handbook on Pædiatric AIDS in Africa by the African Network for the Care of Children Affected by AIDS 2017. Available from: https://www.anecca.org/wp-content/uploads/2018/12/ANECCA-HANDBOOK-2017-ENGLISH-VERSION_20170530_web.pdf.
6. Pugh LE, Roberts JS, Viswasam N, Hahn E, Ryan S, Turpin G, et al. SYSTEMATIC REVIEW OF INTERVENTIONS AIMED AT IMPROVING HIV ADHERENCE TO CARE IN LOW-AND MIDDLE-INCOME COUNTRIES. Journal of Infection and Public Health. 2022.
7. Sefah IA, Mensah F, Kurdi A, Godman B. Barriers and facilitators of adherence to antiretroviral treatment at a public health facility in Ghana: a mixed method study. Hospital Practice. 2022;50(2):110-7.
8. Hudelson C, Cluver L. Factors associated with adherence to antiretroviral therapy among adolescents living with HIV/AIDS in low-and middle-income countries: a systematic review. AIDS care. 2015;27(7):805-16.
9. Rudy BJ, Murphy DA, Harris DR, Muenz L, Ellen J. Patient-related risks for nonadherence to antiretroviral therapy among HIV-infected youth in the United States: a study of prevalence and interactions. AIDS patient care and STDs. 2009;23(3):185-94.
10. Gill MM, Ndimbii JN, Otieno-Masaba R, Ouma M, Jabuto S, Ochanda B. Adherence challenges and opportunities for optimizing care through enhanced adherence counseling for adolescents with suspected HIV treatment failure in Kenya. BMC Health Services Research. 2022;22(1):1-11.
11. MacDonell K, Naar-King S, Huszti H, Belzer M. Barriers to medication adherence in behaviorally and perinatally infected youth living with HIV. AIDS and Behavior. 2013;17(1):86-93.
12. Ajuna N, Tumusiime B, Amanya J, Awori S, Rukundo GZ, Asiimwe JB. Social networks and barriers to ART adherence among young adults (18–24 years) living with HIV at selected primary health facilities of South-Western Uganda: A qualitative study. HIV/AIDS (Auckland, NZ). 2021;13:939.
13. Thompson TL, Haskard-Zolnierek K. Adherence and communication. Oxford Research Encyclopedia of Communication2016.
14. MacPherson P, Munthali C, Ferguson J, Armstrong A, Kranzer K, Ferrand RA, Ross DA. Service delivery interventions to improve adolescents' linkage, retention and adherence to antiretroviral therapy and HIV care. Tropical medicine & international health. 2015;20(8):1015-32.
15. Bervell B, Al-Samarraie H. A comparative review of mobile health and electronic health utilization in sub-Saharan African countries. Social Science & Medicine. 2019;232:1-16.
16. Free C, Phillips G, Galli L, Watson L, Felix L, Edwards P, et al. The effectiveness of mobile-health technology-based health behaviour change or disease management interventions for health care consumers: a systematic review. PLoS medicine. 2013;10(1):e1001362.
17. Creswell JW. A concise introduction to mixed methods research: SAGE publications; 2014.
18. Glasgow RE, Harden SM, Gaglio B, Rabin B, Smith ML, Porter GC, et al. RE-AIM planning and evaluation framework: adapting to new science and practice with a 20-year review. Frontiers in public health. 2019;7:64.
19. Graneheim UH, Lindgren B-M, Lundman B. Methodological challenges in qualitative content analysis: A discussion paper. Nurse education today. 2017;56:29-34.

For peer review only - http://bmjopen.bmj.com/site/about/guidelines.xhtml

20. Abiodun O, Ladi-Akinyemi B, Olu-Abiodun O, Sotunsa J, Bamidele F, Adepoju A, et al. A single-blind, parallel design RCT to assess the effectiveness of SMS reminders in improving ART adherence among adolescents living with HIV (STARTA trial). Journal of Adolescent Health. 2021;68(4):728-36.
21. Pop-Eleches C, Thirumurthy H, Habyarimana JP, Zivin JG, Goldstein MP, De Walque D, et al. Mobile phone technologies improve adherence to antiretroviral treatment in a resource-limited setting: a randomized controlled trial of text message reminders. AIDS (London, England). 2011;25(6):825.
22. Craig P, Dieppe P, Macintyre S, Michie S, Nazareth I, Petticrew M. Developing and evaluating complex interventions: the new Medical Research Council guidance. Bmj. 2008;337.
23. Mehra N, Tunje A, Hallström IK, Jerene D. Effectiveness of mobile phone text message reminder interventions to improve adherence to antiretroviral therapy among adolescents living with HIV: A systematic review and meta-analysis. Plos one. 2021;16(7):e0254890.
24. Tunje A, Jerene D, Kristensson Hallström I. Antiretroviral Therapy and Retention in Care Experiences and Needs of Adolescents Living with HIV in Southern Ethiopia. HIV/AIDS-Research and Palliative Care. 2021:999-1007.
25. McPherson AC, Gofine ML, Stinson J. Seeing is believing? A mixed-methods study exploring the quality and perceived trustworthiness of online information about chronic conditions aimed at children and young people. Health Communication. 2014;29(5):473-82.
26. Spinuzzi C. The methodology of participatory design. Technical communication. 2005;52(2):163-74.
27. Lindgren B-M, Lundman B, Graneheim UH. Abstraction and interpretation during the qualitative content analysis process. International journal of nursing studies. 2020;108:103632.
28. O'Connor C, Leyritana K, Doyle AM, Birdthistle I, Lewis JJ, Gill R, Salvaña EM. Delivering an mHealth adherence support intervention for patients with HIV: Mixed methods process evaluation of the Philippines connect for life study. JMIR Formative Research. 2022;6(8):e37163.
29. Sánchez SA, Ramay BM, Zook J, de Leon O, Peralta R, Juarez J, Cocohoba J. Toward improved adherence: a text message intervention in an human immunodeficiency virus pediatric clinic in Guatemala City. Medicine. 2021;100(10).
30. Mukund Bahadur K-C, Murrayb PJ. Cell phone short messaging service (SMS) for HIV/AIDS in South Africa: a literature review. MEDINFO 2010. 2010:530-4.
31. Lester R, Karanja S. Mobile phones: exceptional tools for HIV/AIDS, health, and crisis management. The Lancet infectious diseases. 2008;8(12):738-9.
32. Hlophe LD, Tamuzi JL, Shumba C, Nyasulu PS. Barriers to anti-retroviral therapy adherence among adolescents aged 10 to 19 years living with HIV in sub-Saharan Africa: A mixed-methods systematic review protocol. Plos one. 2022;17(9):e0273435.
33. Ammon N, Mason S, Corkery J. Factors impacting antiretroviral therapy adherence among human immunodeficiency virus–positive adolescents in Sub-Saharan Africa: a systematic review. Public health. 2018;157:20-31.
34. United Nations Social and Economic Council. Progress towards the Sustainable Development Goals. Report of the Secretary-General. NY. USA; 2022.
35. Creswell JW, Klassen AC, Plano Clark VL, Smith KC. Best practices for mixed methods research in the health sciences. Bethesda (Maryland): National Institutes of Health. 2011;2013:541-5.
36. Lincoln YS, Guba EG. Naturalistic inquiry: sage; 1985.
37. World Medical Association. World Medical Association Declaration of Helsinki: ethical principles for medical research involving human subjects. Jama. 2013;310(20):2191-4.

For peer review only - http://bmjopen.bmj.com/site/about/guidelines.xhtml

For peer review only

For peer review only - http://bmjopen.bmj.com/site/about/guidelines.xhtml

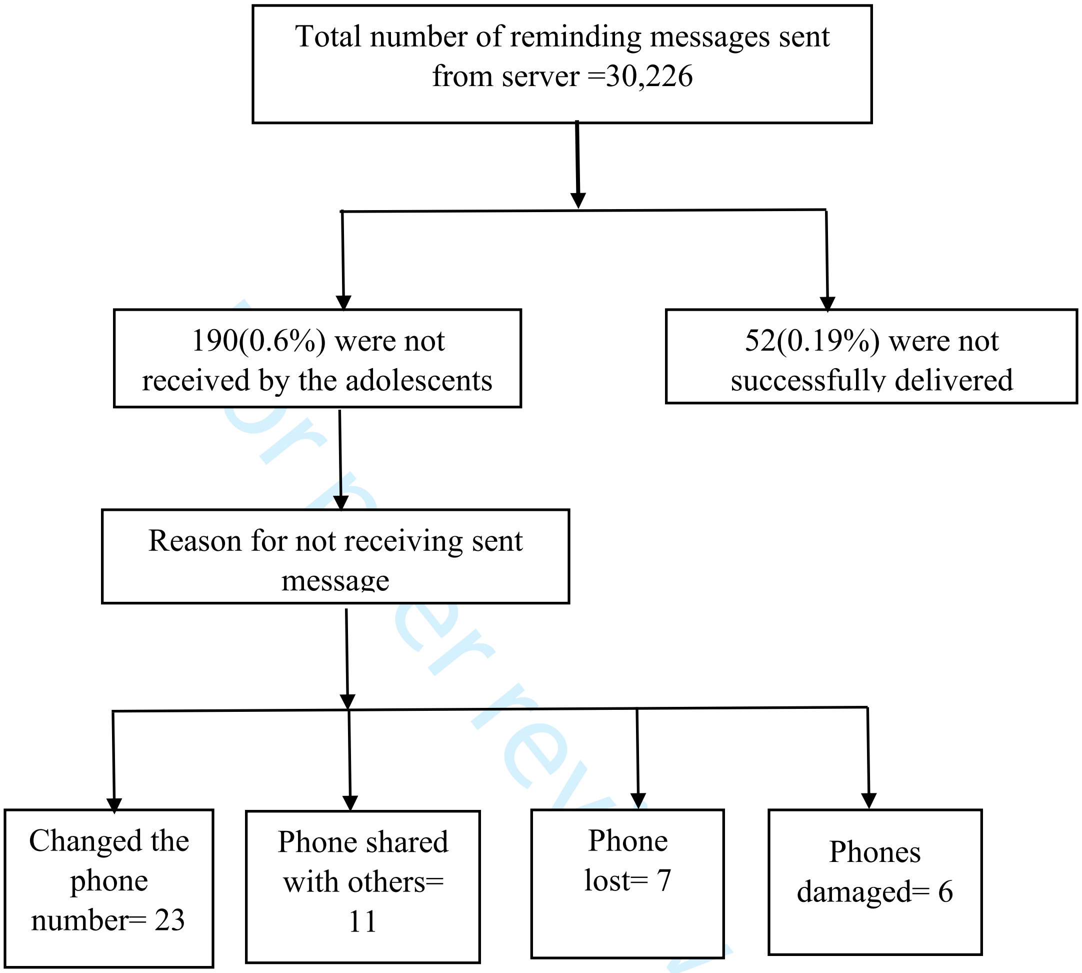

Figure 1. The flow diagram of mobile phone text messaging reminder in the intervention group

BMJ Open

Intervention fidelity and factors affecting the process of implementing a mobile phone text messaging intervention among adolescents living with HIV: a convergent mixed methods study in southern Ethiopia

Journal:	*BMJ Open*
Manuscript ID	bmjopen-2023-077128.R2
Article Type:	Original research
Date Submitted by the Author:	04-Mar-2024
Complete List of Authors:	Tunje, Abayneh; Lund University Faculty of Medicine, Department of Health Sciences; Arba Minch University, Public health<br>Persson, Helene ; Lund University Faculty of Medicine, Department of Health Sciences<br>Jerene, Degu; KNCV Tuberculosis Foundation<br>Hallstrom, Inger; Lunds Universitet,
**Primary Subject Heading**:	HIV/AIDS
Secondary Subject Heading:	Infectious diseases, Public health
Keywords:	Adolescent, HIV & AIDS < INFECTIOUS DISEASES, Information technology < BIOTECHNOLOGY & BIOINFORMATICS

SCHOLARONE™
Manuscripts

I, the Submitting Author has the right to grant and does grant on behalf of all authors of the Work (as defined in the below author licence), an exclusive licence and/or a non-exclusive licence for contributions from authors who are: i) UK Crown employees; ii) where BMJ has agreed a CC-BY licence shall apply, and/or iii) in accordance with the terms applicable for US Federal Government officers or employees acting as part of their official duties; on a worldwide, perpetual, irrevocable, royalty-free basis to BMJ Publishing Group Ltd ("BMJ") its licensees and where the relevant Journal is co-owned by BMJ to the co-owners of the Journal, to publish the Work in this journal and any other BMJ products and to exploit all rights, as set out in our licence.

The Submitting Author accepts and understands that any supply made under these terms is made by BMJ to the Submitting Author unless you are acting as an employee on behalf of your employer or a postgraduate student of an affiliated institution which is paying any applicable article publishing charge ("APC") for Open Access articles. Where the Submitting Author wishes to make the Work available on an Open Access basis (and intends to pay the relevant APC), the terms of reuse of such Open Access shall be governed by a Creative Commons licence – details of these licences and which Creative Commons licence will apply to this Work are set out in our licence referred to above.

Other than as permitted in any relevant BMJ Author's Self Archiving Policies, I confirm this Work has not been accepted for publication elsewhere, is not being considered for publication elsewhere and does not duplicate material already published. I confirm all authors consent to publication of this Work and authorise the granting of this licence.

For peer review only

Intervention fidelity and factors affecting the process of implementing a mobile phone text messaging intervention among adolescents living with HIV: a convergent mixed methods study in southern Ethiopia

Abayneh Tunje, PhD-student[1,2*] abayneh_tunje.tanga@med.lu.se, Helene Åvik Persson, PHN, PhD[1] helene.avik_persson@med.lu.se, Degu Jerene, MD, PhD[3] degu.dare@kncvtbc.org, Inger Kristensson Hallström, RN, PhD, Professor[1] Inger.kristensson_hallstrom@med.lu.se

Affiliations:

[1]Child and Family Health Unit, Department of Health Sciences, Faculty of Medicine, Lund University, Lund, Sweden.

[2] School of Public Health, College of Medicine and Health Sciences, Arba Minch University, Arba Minch, Ethiopia.

[3] KNCV Tuberculosis Foundation, The Hague, Netherlands.

***Corresponding author:**

Abayneh Tunje
abayneh_tunje.tanga@med.lu.se
The Faculty of Medicine
Department of Health Sciences
P.O. Box 157
221 00 Lund, Sweden

Abstract

Objective: To assess the intervention fidelity and explore contextual factors affecting the process of implementing a mobile phone text messaging intervention in improving adherence to and retention in care among adolescents living with HIV, their families, and their healthcare providers in southern Ethiopia.

For peer review only

Design: A convergent mixed-methods design guided by the process evaluation theoretical framework and the RE-AIM framework was used alongside a randomised controlled trial to examine the fidelity and explore the experiences of participants in the intervention.

Setting: Six hospitals and five health centres providing HIV treatment and care to adolescents in five zones in southern Ethiopia.

Participants: Adolescents (aged 10–19), their families and their healthcare providers.

Intervention: Mobile phone text messages daily for 6 months or standard care (control).

Results: 153 participants were enrolled in the process evaluation. Among the 153 enrolled in intervention arm, 78 (49.02%) were male, and 75 (43.8%) were female, respectively. The mean and standard deviation age of the participants is 15 (0.21). The overall experiences of implementing the text messages reminder intervention were described as helpful in terms of treatment support for adherence but had room for improvement. During the study, 30,700 text messages were sent, and fidelity was high, with 99.4% successfully delivered text messages during the intervention. Barriers such as failed text messages delivery, limitations in phone ownership, and technical limitations affected fidelity. Technical challenges can hinder maintenance, but a belief in the future of digital communication permeates the experiences of the text messages reminders.

Conclusions: Overall fidelity was high, and participants' overall experiences of mobile phone text messages were expressed as helpful. Contextual factors, such as local telecommunications networks and local electric power, as well as technical and individual factors must be considered when planning future interventions.

Trial registration: Pan African Clinical Trials **PACTR202107638293593, first registration** 20/07/2021.

Strengths and limitations with this study

For peer review only - http://bmjopen.bmj.com/site/about/guidelines.xhtml

- Secure and confidential delivery of high-volume messages through a 4-digit short code.
- The use of a mixed methods design and the inclusion of family members and healthcare providers is a strength of this process evaluation study.
- There was a limited number of adolescents´ family and healthcare providers included in the interviews.

INTRODUCTION

Globally, 38.4 million people were living with HIV in 2021. Of these, 1.71 million were adolescents aged 10–19, 88% of whom live in Sub-Saharan Africa (1, 2). To control the epidemic, the Joint United Nations Programme on HIV/AIDS (UNAIDS) launched the 95%–95%–95% targets aimed at HIV testing, treatment, and viral suppression by 2030 (3). However, by 2021, 75% percent of the 38.4 million people living with HIV (PLHIV) were receiving antiretroviral therapy (ART), a treatment gap of 5.9 million people (4). Moreover, achieving the recommended optimal adherence level of >95% which is required for ART to be effective, remains a challenge within the HIV care continuum (5). This poses a significant threat to HIV interventions in resource-limited settings (6).

Poor adherence to ART has several consequences, including increased risk of viral drug resistance and reduced treatment effectiveness toward viral suppression, leading to disease progression, greater risk of death, and increased risk of viral transmission (7). Some of the major barriers to ART adherence include socioeconomic status,(8, 9) fear of being stigmatised or discriminated against as a result of one's HIV status,(10) forgetting to take medication on time,(11) treatment fatigue,(12) and patient–provider communication (13). Several interventions to overcome these barriers have been studied in a variety of settings (14). The use of digital health strategies to enhance HIV treatment is one of the priority interventions in Sub-Saharan Africa (15). Targeted

For peer review only - http://bmjopen.bmj.com/site/about/guidelines.xhtml

digital client communication using mobile phone-based short text messaging has been recommended for adherence support for a range of health issues and adherence to antiretroviral treatment (16). The success and effectiveness of an mHealth intervention in achieving desired health outcomes is dependent on the context in which they are implemented (15, 16). Context plays a crucial role in either the success or failure of an intervention.

In addition to design and implementation, the success of interventions is diverse and may be influenced by factors such as sociodemographic and culture (17). Observing what is delivered in practice with close reference to the theory of the intervention can help evaluators in distinguishing the intervention fit in different contexts and changes that undermine intervention fidelity (15, 17). Trials should continue to rigorously assess outcomes, but also include integral process evaluations that use qualitative and quantitative data to develop and test hypotheses about how interventions work (18). In a multi-centre trial, a process evaluation is necessary to understand whether the intervention was implemented and received similarly across all sites (9). However, trials assessing mHealth adherence interventions for HIV often do not include process evaluations to examine the fidelity and quality of the intervention delivery, causal mechanisms for the health outcomes, contextual factors affecting the delivery, and costs of implementation (16, 19). Understanding the entire implementation process of an mHealth intervention allows practitioners to interpret the results and replicate the intervention in other contexts (20, 21). Therefore, this study aimed to evaluate the process of a mobile text messaging intervention conducted among adolescents living with HIV in Ethiopia.

METHODS

Study design

For peer review only - http://bmjopen.bmj.com/site/about/guidelines.xhtml

A convergent mixed-methods design (17) combining quantitative and qualitative techniques was used. The design was guided by the process evaluation theoretical framework (22) and structured by the RE-AIM dimensions (Reach, Effectiveness, Adoption, Implementation and Maintenance) (18) to evaluate intervention effectiveness alongside a randomised controlled trial (RCT) to examine the study's fidelity and to explore the experiences of the participants in the intervention.

The trial was registered in Pan African Clinical Trials Registry with the registration number PACTR202107638293593 on 17/07/2021.

Study setting and participants

Six hospitals and five health centres that provide HIV treatment and care to adolescents in the Gamo, Gofa, Konso, South Omo, and Wolayita zones in Southern Ethiopia were included in the main study. These zones are home to over 25 distinct ethnic groups, each with its own culture and context. Between July 5, 2022, and February 28, 2023, 306 adolescents living with HIV were included in the study, in either the intervention or in the control group. The quantitative data included process indicators, while the qualitative interviews included ten adolescents from the main trial, four adolescents' families, and two healthcare providers who were involved in the process evaluation. We used the following technique to identify eligible adolescents: first, we identified the number of adolescents of eligible age in each facility. Then, using their address, we called each adolescent and their parents or caregivers directly via phone numbers, care providers, or adherence supports. Then, data collectors approached adolescents and posed literacy questions about their ability to read text by displaying sample texts. Then, eligible participants in the study were asked to give consent and assent. Thus, adolescents aged 10 to 19 years, diagnosed with HIV, currently participating in ART care, and planning to stay in the designated facility for at least six months following study enrolment were eligible. Participants who were above the age limit, did not

For peer review only - http://bmjopen.bmj.com/site/about/guidelines.xhtml

disclose their HIV status, were unable to read text, or had a disability were all excluded. Finally, the data collector showed each eligible adolescent how to use their mobile device, including text saving and deletion.

Theoretical Framework of Process Evaluation

The RE-AIM framework (18) was used for the process evaluation to comprehensively examine the level of implementation. This involves understanding the implementation of interventions in terms of (1) fidelity, defined as to what extent the intervention was implemented consistently with the underlying theory as planned; (2) delivery, defined as to what extent all of the intended activities, training, and materials were provided to program participants; (3) reach, defined as the absolute number, proportion, and representativeness of individuals who are willing to participate in a given initiative, intervention, or program; (4) receipt, defined as how participants reacted to specific aspects of the intervention; and (5) context, defined as what contextual factors influence implementation or the intervention outcome.

Description of the intervention

The MRC framework,(22) a systematic literature review,(23) interviews with adolescents,(24) and the theoretical health belief model (HBM) (25) were used to develop the intervention. Adolescents living with HIV, healthcare providers, and research project technical advisory team members participated in the design (26) under the guidance of a research advisory committee. The HBM is a theoretical model that has been constructed from six domains: susceptibility, severity, barriers, benefit, cues to action, and self-efficacy. After the intervention's development, text messages were uploaded to the server, and an automated message-pushing software was used to send tailored daily text messages using a four-digit code from Ethiopia Telecom. This code helps adolescents in

For peer review only - http://bmjopen.bmj.com/site/about/guidelines.xhtml

avoiding messaging source-related stigma and distinguishing intervention messages from other messages.

Other various configurations such as wide-area network (WAN) internet protocol (IP), local area network (LAN) IP, Ethio telecom central server IP, a virtual private network (VPN) line, and office servers were used as infrastructure, as required to support the study. The text messages were categorised and delivered based on each adolescent's customised medication schedule and time. The software sent the message 15 minutes before each participant's medication time. The type of message was short and mainly focused on advice about the benefits of taking on time, the risk/consequences of missing/not taking on time, encouragement, and without mentioning anything about HIV or the name of the drug. For example, “Those who use it properly know its benefits”, “I'm taking it properly and on time”. For example, these messages in Amharic are “በትክክል የሚጠቀሙት ጥቅሞቹን ያውቃሉ) and በትክክል እና በሰዓት እወስዳለሁ respectively. The server was checked daily to troubleshoot network/cell phone issues. Based on the server information, the first author contacted the facility level care provider to contact the adolescent with corrective actions; for example, subscriber identity module card (SIM card) replacement, damaged mobile phones, and network troubleshooting for maintenance and replacement.

Sample and data collection

The quantitative data was collected from the server, activity logs, an adverse event monitoring form, and an interview with adolescents. The researcher collects data on intervention fidelity by tracking the number of text messages sent, delivered, and failed from the server daily.
Furthermore, each facility representative documented any adverse event related to the trial, filled the form, and sent it to researchers every week. Furthermore, during the follow-up data collection phase, adolescents asked if they received text messages on a regular schedule.

For peer review only - http://bmjopen.bmj.com/site/about/guidelines.xhtml

A purposive sampling of 10 adolescents with HIV (aged 12–19), four family members/relatives (aged 36–45), and two healthcare providers (aged 31–36) from five different healthcare facilities were asked to be interviewed about their experiences in the intervention between July 5, 2022, and February 28, 2023, at healthcare facilities by the first author. The interviews were conducted in all respondent languages, such as Amharic, Wolaytigna, Gamogna, Gofigna, Arigna, and Konsegna in a quiet and confidential area at the respective health facilities by the first author and one research assistant and lasted on average 30 minutes. All interviews were audio-recorded with the permission of the participants.

An interview guide was used to gain a rich, in-depth understanding, and to allow the participants to concretise their experiences of their use of text messages, communication, and technical aspects of the intervention. The interview guide included subsections adapted to the participants, i.e., adolescents, families, and healthcare providers. Hence, the questions were different, depending on to whom they were addressed. The introductory question to the adolescents was: "Are you receiving the mobile text-message reminder from the hospital?", followed by follow-up questions such as, "What do you think about the messages?" The families were initially asked "How do family/social related factors affect ART adherence among adolescents?" Healthcare providers were asked "How did you experience communication with the adolescents with regard to the intervention?" Follow-up questions and prompts were used throughout the whole interview.

The interviews were audiotaped, transcribed verbatim, and translated from Amharic to English by the first author, and checked for accuracy by one independent language expert.

Data analysis

The number of SMS messages sent and delivered to participants were tracked as a measure of intervention fidelity. When SMS messages did not arrive on the phone, the project advisory team

For peer review only - http://bmjopen.bmj.com/site/about/guidelines.xhtml

investigated the external network and other infrastructure issues. If the message could not be delivered during this period, the computer automatically recorded it as a delivery failure and sent the message again. Data captured on the delivery status of the SMS messages were recorded as: delivered (the phone had reception marked as green); undelivered (the phone was switched off or marked as red in the server). The program could not record whether messages delivered to the phones were opened.

The descriptive statistical analysis was used to summarize data in frequency, percentages, mean and standard deviation. The quantitative effectiveness data is analyzed separately and not included in the process evaluation. The RE-AIM outcomes framework components of Reach, Effectiveness, Adoption, Implementation, and Maintenance were used to present mixed method findings about the delivery of a reminder message, success, and intervention barriers for the intervention implementation process (18).

Interviews were analysed by using the principles for content analysis described by Graneheim and Lundman (19, 27). First, the transcripts were read several times to gain a sense of the whole by the first and the last author. Thereafter, meaning units were identified and condensed to reduce the text while maintaining the core meaning. The first author developed a coding frame for the analysis by using NVivo software, which were discussed by all authors. Condensed meaning units were then labelled with a code, which were kept close to the text on a manifest level. Next, sub-themes were created from the code frame by the first and last authors. The sub-themes were then abstracted into themes. Finally, the underlying meaning—the latent content—was described into a main theme. Thereafter one of the authors (HÅP) read the transcripts, extracted meaning units, codes, and themes. The content was discussed and reflected upon together with all authors and lasted until an agreement was reached.

For peer review only - http://bmjopen.bmj.com/site/about/guidelines.xhtml

207 **Patient and Public Involvement**

208 Representatives of adolescents were involved in the development of messages for the SMS
209 reminders and interview guides. Their feedback was used to adapt its contents and to set strategies
210 to promote inclusivity in study participation during participant recruitment. In addition,
211 participants, healthcare providers, and the participants' families were involved in reporting any
212 incidence related to mobile devices and other issues.

213 **RESULTS**

214 **The characteristics of adolescents assigned in the intervention arm are shown in table 1.**

215 Table 1. Participant characteristics

Characteristics of adolescents assigned in the intervention		
**Characteristics**		Frequency /Mean (SD)/ Percent
Adolescent age, Mean (standard deviation (SD))		15(0.21) years
Adolescents age category	10-14	60 (39.22)
	15-19	93 (60.78)
Adolescents gender	Male	78 (49.02)
	Female	75 (43.8)
Languages of adolescent during interview	Amharic	62 (40.52)
	Wolaytigna	27 (17.65)
	Gamogna	39 (25.49)
	Gofigna	13 (8.5)
	Arigna	6 (3.3)
	Konsegna	6 (3.3)
Educational status of adolescent	Grade 1-8	97 (63.40)
	Grade 9 and above	56 (36.60)
Residence of adolescent	Urban	111(72.55)
	rural	42 (27.45)
Living arrangement	Live alone	10 (6.54)
	Live with family	143 (93.46)
Psycho-social peer-support	Participate	47 (30.72)
	Do not Participate	106 (69.28)
Interviewee characteristics		
Adolescents (Sex/age)	Male (10-14 years)	2
	Female (10-14 years)	3

For peer review only - http://bmjopen.bmj.com/site/about/guidelines.xhtml

	Male (15-19 years)	2
	Female (15-19 years)	3
Adolescents level of education	Grade 1-8	5
	Grade 9 and above	5
healthcare providers (Sex)	Male	1
	Female	1
Parent/care giver (Sex)	Non-biological care giver	2 (1 male and 1 female)
	Biological	2 (1 mother and 1 father)

The results are structured using the RE-AIM framework (18) and corresponding themes (Table 2).

Table 2. Overview of the qualitative themes related to the dimensions in the RE-AIM framework.

**RE-AIM dimension (18)**	**Definition (18)**	**Qualitative theme**
Reach	The number, proportion, and representativeness of eligible individuals who participate in each initiative.	
Effectiveness	The impact of an intervention on the relevant outcomes, including potential adverse effects, quality of life, and economic outcomes.	Text message reminders were helpful for adherence but there was room for improvement
		Text messages reminders support adherence
		Feeling comfortable receiving text messages reminders
		The code delivered with text messages reminders minimises the risk of stigma.
Adoption	The reach and effectiveness/efficacy of an intervention at the setting level.	Supporting the text messages reminders
Implementation	The intervention agents' fidelity to the various elements of an intervention's protocol, including consistency of intended delivery.	Failing text messages delivery influences medication intake
		Limitations in phone ownership
		Technical limitations

For peer review only

For peer review only - http://bmjopen.bmj.com/site/about/guidelines.xhtml

Maintenance	Connects with both setting-level indicators (the extent to which a program or a policy becomes part of organisational practices and policies), and individual-level indicators (monitoring of effectiveness of an intervention or program six months or more after the most recent contact).	Seeing potential in SMS services
		Possibilities of text messages reminders

Reach:

The total number of individuals that were assessed for eligibility was 435, of which 306 (70.34 %) meet eligibility criteria. The remaining 129 participants excluded for the following reasons: 27 (20.9%) were unable to stay in the study area for the duration of the study, 39 (30.2%) were unable to read, 24 (18.6%) were did not know their status, 21 (16.3%) were could not reached, 13 (10.1%) refused to participate, and 5 (3.9%) had an impairment. Thus, 306 participants were enrolled and randomised: n =153 in the intervention, and n =153 in the control group. Among the 153 enrolled in intervention arm, 78 (49.02%) were male, and 75 (43.8%) were female, respectively. The mean and standard deviation age of the participants is 15 (0.21). Of these, 111 (72.55%) resided in an urban area, 97 (63.40%) had primary school level education, and 10(6.54%) live alone(orphan).

Effectiveness:

The number of participants retained in the 6-month follow-up of the intervention group was higher than the control group in this study.

Text messages reminders were helpful for adherence but there was room for improvement

The overall experiences of the intervention among adolescents living with HIV was that text messages reminders were helpful when it came to their adherence to medication, but direct access to phones and better timing of messages should be assured to increase their functioning and

For peer review only

For peer review only - http://bmjopen.bmj.com/site/about/guidelines.xhtml

usability. The retention rate was improved, and was the best achieved since the intervention was introduced. Despite this, more effort was requested concerning technical issues to make the reminders even more helpful. The participants described facilitating aspects like increased medication intake and communication. Also, the well-being of the adolescents and the support function was highlighted. However, technical problems like lost telephones or text messages reminders arriving after medication time were emphasised as barriers.

Text messages reminders support adherence

They described that before the intervention, their medication was often forgotten, which resulted in it being taken later than it should have been due to forgetfulness. Sometimes the adolescent realised that they had forgotten to take their medication but then waited until dinner to take it. The adolescents described that text messages reminders were associated with taking their medicine and delays were avoided. Also, family members described situations when the adolescent's medication routines were interrupted, and where text messages reminders played a big role.

> *"…Yes, it supports treatment adherence, because most of time we follow the television program but sometimes the power goes off, we missed the exact time to see medication time. Currently this text message comes 10 to 15 minutes before our medication time."*
> *(Father, aged 45)*

Feeling positive about the text messages reminder

The text messages reminders contributed to positive attitudes and to decreased stigmatisation for adolescents, even when HIV/AIDS stigma is deeply rooted in the culture. Thus, the perception of the adolescents was that the text messages reminders played an important role for their overall well-being, because sometimes they had low moods. The text messages reminders were described

For peer review only - http://bmjopen.bmj.com/site/about/guidelines.xhtml

as offering hope and supported the adolescents in achieving consistency in their medication adherence which helped them avoid, for example, fatigue.

"…The benefit of this message is it comes always and reminds me to take the drug on time. The other benefit is when I receive the message, I feel good." (Adolescent, aged 13)

The code-delivered text messages reminders minimise the risk of stigma

The adolescent felt safe regarding privacy in the text messages reminders due to a code sent out to their telephone. The healthcare provider noticed a change in the views of both adolescents and family regarding increased trust and improved engagement with healthcare facilities during the intervention. Family expressed receiving coded text messages reminders could enable adolescents to live like other HIV-free adolescents. However, the adolescents indicated that after their family's HIV status was made public, they were subjected to stigma, which was reflected in the interview. Some members of the families were open with their HIV disclosure while others were not.

"My status has already been made public, and everybody knows about my family as well ... However, my husband lives in another area because of work, and knows his HIV status and makes things secret. He did not even take his medication when he come back home." (Mother, aged 45)

Adoption:

In this study, eleven health facilities with 153 participants received an intervention across 5 different geographic areas. According to healthcare providers, the rate of retention among adolescents has increased since the study began in the hospital, as described in the qualitative findings under a sub-theme.

For peer review only - http://bmjopen.bmj.com/site/about/guidelines.xhtml

".... By the way, in terms of retention, we have observed the best retention rate ever in this year since ART started in this hospital. Of course, our follow-up is there and there are other partners like CDC and ICAP. But this messaging has augmented the service we provide, and it increased our communication with them and helped them remind their medication irrespective of inconsistencies in messaging and medication time; the message delivery reminds them something targeted." (healthcare provider, aged 36)

Supporting the text messages reminders

The healthcare providers emphasised that text messages reminders were an important support for adolescents in their medication intake to avoid poor adherence. In addition to this, families indicated that they had a mission to support the adolescents in avoiding ART treatment fatigue. Several adolescents expressed that they had received support from their family concerning medication intake during the intervention. Thus, the text messages reminders were perceived as helpful, as they made participants feel happy and hopeful, even if they felt angered or were in a depressed mood that day.

"...The messages may help people pass good days, despite days that do not carry good fortune. It therefore helps people have good days and reminds them of medication times. One day, for example, being consumed with some ideas, I did not recall that I have medication to take; it is this text message that triggered me take my medicine." (Adolescent, aged 19)

Implementation:

There were 306 mobile phones provided to adolescents during assignments into intervention and control groups. The advisory committee was involved in the implementation of the intervention using standard operational procedure (SOP). During the study, 30,700 SMS text messages were sent to 153 study participants in the intervention group (Figure 1).

For peer review only - http://bmjopen.bmj.com/site/about/guidelines.xhtml

Overall, intervention fidelity was high with (30,510/30,700) or 99.38% successfully delivered SMS text messages during the intervention.

The non-receiving participants in the intervention group were tracked and cross-checked using individual participant codes from the server, and then approached by each health facility to confirm whether they were to be followed-up.

Changing phone numbers/SIM cards, phones being damaged, lost, or shared with others were identified as participant-related reasons for not receiving the messages, and were registered in the logbooks, and corrective measures were used either to replace phones with new devices or repair/maintain the phones as soon as was feasible.

Failing text message delivery influences medication intake

The adolescents identified several reasons for not receiving messages. In connection with the technical aspect, the adolescents highlighted problems such as not having a charged phone battery or having lost their phone. During the feasibility study period, there was fluctuation in messaging time, and the text message reminders arrived after their medication time. Another issue observed by both healthcare providers and family members was a missing SIM card. Furthermore, the continuity in the text messages reminders was requested by the adolescents to continue the benefits.

> *"… the receiving time varies; sometimes I receive it 10 minutes before medication time, which is 3p.m. local time in the evening, and sometimes it arrives after I take the drug." (Adolescent, aged 13)*

Limitations in phone ownership

Sometimes adolescents were not permitted to use the telephone by themselves, due to restrictions from their family, as they thought that the adolescents were too young to use the telephone.

For peer review only - http://bmjopen.bmj.com/site/about/guidelines.xhtml

Instead, the family used the telephone, which resulted in text messages reminders never reaching the adolescent. The absence of SIM cards may be because adolescents did not receive a SIM card, or because someone else in the family was using them. The family reported that they passed information from healthcare providers to the adolescents. Some of the families lacked information about the intervention and did not know what to do when the telephone arrived. The healthcare providers emphasised that some families denied the adolescents use of the telephone, which could cause problems, and text messages reminders were not received. Furthermore, the importance of informing families about the intervention was stressed, as several adolescents were restricted from using the telephone.

"…I think families should be communicated with regarding why their children are provided with mobile phones from the hospital, so as to avoid denial to access or adults using the phones for themselves." (Healthcare provider, aged 36)

Technical limitations

One major issue stressed by adolescents, families, and healthcare providers, was that the electric power system went off quite often in their communities, which caused problems with receiving text messages reminders if the battery in the telephone was not charged. The adolescents usually did not experience problems with delays in text messages reminders, but they had observed that the text messages reminders did not always arrive on time. The messages could come long before their scheduled medication time or sometimes very close to or after their scheduled medication time. Sometimes the text message reminder was absent for entire days.

"…The message helped me a lot to remember my medication time. However, the skips in message delivery dates and untimely arrivals should be corrected so that it is more helpful." (Adolescent, aged 18)

Maintenance:

For peer review only - http://bmjopen.bmj.com/site/about/guidelines.xhtml

After initial configuration, a single server can send text messages to thousands of participants across a large geographic area. In this study, a server sent text messages to 153 participants across five different geographic areas. The technical challenges experienced in delivering the intervention were related to navigating the messaging server and feedback responses from the participants. The non-receiving participants in the intervention group were tracked and cross-checked using a four-digit individual participant codes from the server and were then approached by each facility to confirm whether they were indeed in that group or lost. Despite technical barriers, and adverse conditions, and a belief in the future of text messages reminders emerged, which was also expressed in the qualitative interviews.

Seeing potential in SMS services

The benefits of SMS reminders were perceived as valuable; especially when adolescents missed appointments for medication refills and virus load tests. Before the intervention, there were difficulties getting in touch with the adolescents, because they lived in rural areas and did not have a telephone. The healthcare providers thought about how to incorporate text messages reminders into routine health care. A wish among the adolescents was expressed that more text messages reminders could be helpful in raising awareness about their condition. Both the adolescents and the healthcare providers raised a need to continue the text message reminders.

> *"I think this must continue. The mobile provision has played significant roles in adherence and retention, as many of the adolescents do not have phones…because of this provision, adolescents can be reached directly with health messages and receive important lessons just from their phones." (Adolescent, aged 19)*

Possibilities with text messages reminders

For peer review only - http://bmjopen.bmj.com/site/about/guidelines.xhtml

The healthcare provider described that possibilities to communicate with the adolescents had increased after the intervention, and the adolescents' attitudes towards healthcare facilities had become more positive. The adolescents found the improved digital communication to be an advantage because the healthcare provider no longer needed to call their family but could call them directly.

"Previously, healthcare providers from hospital reached me through neighbourhood phone numbers. She now uses this number to reach me directly through the phone provided by the hospital. This is a benefit for my privacy and for the information we discuss over the phone." (Adolescent, aged 18)

DISCUSSION

Our process evaluation which was based on the RE-AIM framework (18) showed that intervention fidelity was high with only about 0.6% of the SMS messages not delivered as intended. Both providers and patients described the SMS reminders as being helpful. A few barriers were noted including glitches at telecommunication networks, electric power disruptions, issues with phone ownership at household levels. Our results highlight that SMS message reminders can be delivered with high coverage if proper processes are followed. The 4-digit short code messaging system was a key enabler in ensuring confidentiality and security of the messages. Further scale-up of such interventions will depend on addressing broader systems issues including phone ownership, fixing network interruptions and reliable power sources. However, a study conducted in Philippines showed that intervention fidelity was low (77.9%) in the message group. The identified reason for low intervention fidelity was poor reliability of local telecommunication networks, and frequency of messages received (28). The local context for cellular phone infrastructure and operational challenges, such as multiple users on a single cell phone, has an impact on text messaging interventions (29). It is important to assess the setting before using the SMS intervention as a strategy to improve adherence. There is emerging evidence that mobile phones can play an

For peer review only - http://bmjopen.bmj.com/site/about/guidelines.xhtml

important role in healthcare delivery, especially in resource-limited settings (30). SMS text messaging is a particularly useful application that can be used to collect or share information and to enhance communication between healthcare personnel and patients in a low-cost manner (31). Treatment support was highlighted as an important factor for adolescents to avoid poor adherence. Text messages could be a facilitator for adolescents to remain hopeful and avoid unwanted side effects given that previous research (32, 33) has shown that a lack of support systems was perceived to negatively affect adherence among young people. Our findings provide additional evidence in support of research to identify effective interventions to improve adherence and retention in care for adolescents with HIV and enhance their engagement in these services. Thus, findings of this process evaluation can guide further research towards interventions concerning readiness among healthcare facilities, because settings can impact implementation. Giving importance to the voice of adolescents living with HIV can offer hope to many more in the same situation. This study thereby has a potential clinical impact and contributes to UN Sustainability Goal (34).

Methodological concerns/ limitations

There are both strengths and limitations identified in this process evaluation. First, the intervention has several strengths, and a key strength is the use of a 4-digit short code for text messages that enables rapid, high-volume outbound messaging, is easy to read and remember, and assists text recipients in identifying the sender based on their preferences for message delivery time, message content, and messaging language. The use of the unique code can contribute to maintaining security and confidentiality among participants.

Secondly, the messaging server is adaptable and user-friendly, as the researcher or local IT provider can add and remove new data fields to navigate dropped or missed individuals and update SMS text message content to avoid message-related fatigue.

For peer review only - http://bmjopen.bmj.com/site/about/guidelines.xhtml

In addition, the process evaluation also has several strengths that were identified. The first strength is the use of mixed methods, where different forms of data—both quantitative and qualitative methods—are combined to capture the multi-dimensionality of the intervention. The data supplement one another and aid in obtaining a comprehensive picture of the study results, which can contribute to a deeper understanding of the participants' experiences (35). If the participants are given the opportunity to highlight their own individual priorities, it might be possible for us to improve our understanding of the impact of the intervention.

Finally, three different perspectives (adolescents, families, and healthcare providers) were included in the qualitative interviews, which allowed different voices to be heard. However, limited numbers of included participants from adolescents' families and healthcare providers can limit the study's transferability (36). The way research results are presented can be influenced by their nature and outcome, leading to what is known as reporting bias. To minimize outcome reporting bias, the study protocol was pre-registered in the pan-African clinical trials registry. A composite adherence measurement was used, and CONSORT recommendations were followed (37). The fact that adherence data was self-reported by healthcare professionals within the same facilities through interviews may have a significant impact on the study's validity and make it prone to social desirability bias. However, in this study, the primary author conducted the interviews in a separate, private room. Furthermore, providing cell phones to adolescents during recruitment may reduce the likelihood of interview refusal in the intervention phase, causing acquiescence bias.

Trustworthiness is the most commonly used criterion for evaluating qualitative content analysis, and it is often expressed using terms such as credibility, conformability, dependability, and transferability (36). To ensure the credibility of the study, an appropriate data collection method needs to be used(38). This study used separate interview questions for adolescents, family, and

For peer review only

For peer review only - http://bmjopen.bmj.com/site/about/guidelines.xhtml

professional interviews to explore their experiences and issues with mobile text messaging interventions. In this study, the five-dimension components of the RE-AIM framework were used to collect information about the intervention setting, implementation, personnel and circumstances, and findings by considering individual level and external context challenges(18). To ensure the dependability of the study, an interview guide adapted to the participants, and audio-recorded was used. For the confirmability of this study, the first author transcribed the audio data to verbatim text and translated from Amharic to English by considering both manifest and latent content. Then, all authors involved in reading the transcripts, extracted meaning units, codes, and data analysis.

Finally, the authors previous qualitative research experiences and knowledge, as well as a bracketing technique were used to increase the trustworthiness of their findings. Accordingly, the primary author maintained a positive relationship with participants during intervention follow-ups and established independence from caregiving facilities to minimize social desirability bias and boss views. To reduce bias and subjective interpretation, interviews were audio recorded, transcribed, and thoroughly evaluated, with participant quotes used to support the authors' findings.

Conclusion

The mobile phone text messaging intervention to support ART adherence and retention in care was well-received by participants, and overall intervention fidelity was high. However, the intervention's feasibility was dependent on the reliability of local telecommunication networks, local electric power, and monitoring of adolescents' families, all of which had a significant impact on the intervention's usability, fidelity, and doses received. The participants experienced the text messaging function as helpful, therefore the development of different aspects, such as addressing technical problems, would be desirable to further improve impact. Further research needs to be

For peer review only - http://bmjopen.bmj.com/site/about/guidelines.xhtml

directed toward readiness within the healthcare facilities to capture an increased understanding of barriers and facilitators in implementation.

Author affiliations

[1]Child and Family Health Unit, Department of Health Sciences, Faculty of Medicine, Lund University, Lund, Sweden.

[2] School of Public Health, College of Medicine and Health Sciences, Arba Minch University, Arba Minch, Ethiopia.

[3] KNCV Tuberculosis Foundation, The Hague, Netherlands.

Acknowledgements

The authors sincerely thank the participants for sharing their experiences, and all staff working in the ART clinics for facilitating the study.

Contributors

AT, DJ, and IKH conceptualized and developed the study design. AT collected the data and transcribed verbatim and translated from Amharic to English. DJ and IKH supervised data collection and study implementation. All authors read the transcripts, extracted meaning units, codes, and data analysis. Sub-themes were created from the code frame by the first and last authors. AT and HÅP drafted the manuscript, and all authors contributed to critical revision and approved the final manuscript.

Funding

The project was externally funded by FORTE: Swedish Research Council for Health, Working Life and Welfare Life (FORTE) (https://forte.se/en/) program support 2018–01399 and the

For peer review only

Swedish Research Council(https://www.vr.se/english.html) program support VR 2016-05706. The study protocol has undergone a peer review by the funding body. The funders had no role in study design, writing the study protocol, the decision to publish the study protocol, or preparation of the manuscript.

Competing interests

None declared.

Patient and public involvement

Patient consent for publication

Not applicable.

Ethics approval

The study was conducted according to the guidelines in the Declaration of Helsinki (39) and approved by the Swedish Regional Ethical Review Board (Dnr 2019-03433), National Research Ethics Review Committee in Ethiopia (MoSHE/RD/142/2869/20), and the Institutional Research Ethics Review Board in Arba Minch University (IRB-113/11). The participants received both oral and written information about the purpose of the study, about the confidential treatment of the data and the voluntary nature of participation. Informed consent was provided from all participants over the age of 18 before the study began. Similarly, parental consent and adolescent assent were obtained from all participants under the age of 18 before the start of the trial.

Provenance and peer review

Not commissioned, externally peer reviewed.

Data availability statement

For peer review only - http://bmjopen.bmj.com/site/about/guidelines.xhtml

No datasets were generated or analysed during the current study. All relevant data from this study will be made available upon reasonable written request and in accordance with ethical approval, from the corresponding author.

Supplemental material

Open access

This is an open access article distributed in accordance with the Creative Commons Attribution Non-Commercial (CC BY-NC 4.0) license, which permits others to distribute, remix, adapt, build upon this work non-commercially, and license their derivative works on different terms, provided the original work is properly cited, appropriate credit is given, any changes made indicated, and the use is non-commercial. See: http://creativecommons. org/licenses/ by-nc/ 4.0/

ORCID

Abayneh Tunje (orcid.org/0000-0002-0957-0216)
Degu Jerene (orcid.org/ 0000-0001-7853-4211)
Helene Åvik Persson (orcid.org/0000-0002-7862-5444)
Inger Kristensson Hallström (orcid.org/0000-0002-9402-6230)

References

1. UNAIDS. Global HIV & AIDS statistics — Fact sheet: https://www.unaids.org/en/resources/fact-sheet. 2021.
2. UNICEF. Global and regional trends. 2022 [Available from: https://data.unicef.org/topic/hivaids/global-regional-trends/.
3. Duncombe C, Ravishankar S, Zuniga JM. Fast-Track Cities: striving to end urban HIV epidemics by 2030. Current Opinion in HIV and AIDS. 2019;14(6):503-8.
4. World Health Organization. HIV data and statistics. World Health Organization (WHO) Available online: https://www who int/teams/global-hiv-hepatitis-and-stis-programmes/hiv/strategic-information/hiv-data-and-statistics. 2021.
5. African Network for the Care of Children Affected by HIV/AIDS – ANECCA. Handbook on Pædiatric AIDS in Africa by the African Network for the Care of Children Affected by AIDS 2017. Available from: https://www.anecca.org/wp-content/uploads/2018/12/ANECCA-HANDBOOK-2017-ENGLISH-VERSION_20170530_web.pdf.

For peer review only - http://bmjopen.bmj.com/site/about/guidelines.xhtml

6. Pugh LE, Roberts JS, Viswasam N, Hahn E, Ryan S, Turpin G, et al. SYSTEMATIC REVIEW OF INTERVENTIONS AIMED AT IMPROVING HIV ADHERENCE TO CARE IN LOW-AND MIDDLE-INCOME COUNTRIES. Journal of Infection and Public Health. 2022.
7. Sefah IA, Mensah F, Kurdi A, Godman B. Barriers and facilitators of adherence to antiretroviral treatment at a public health facility in Ghana: a mixed method study. Hospital Practice. 2022;50(2):110-7.
8. Hudelson C, Cluver L. Factors associated with adherence to antiretroviral therapy among adolescents living with HIV/AIDS in low-and middle-income countries: a systematic review. AIDS care. 2015;27(7):805-16.
9. Rudy BJ, Murphy DA, Harris DR, Muenz L, Ellen J. Patient-related risks for nonadherence to antiretroviral therapy among HIV-infected youth in the United States: a study of prevalence and interactions. AIDS patient care and STDs. 2009;23(3):185-94.
10. Gill MM, Ndimbii JN, Otieno-Masaba R, Ouma M, Jabuto S, Ochanda B. Adherence challenges and opportunities for optimizing care through enhanced adherence counseling for adolescents with suspected HIV treatment failure in Kenya. BMC Health Services Research. 2022;22(1):1-11.
11. MacDonell K, Naar-King S, Huszti H, Belzer M. Barriers to medication adherence in behaviorally and perinatally infected youth living with HIV. AIDS and Behavior. 2013;17(1):86-93.
12. Ajuna N, Tumusiime B, Amanya J, Awori S, Rukundo GZ, Asiimwe JB. Social networks and barriers to ART adherence among young adults (18–24 years) living with HIV at selected primary health facilities of South-Western Uganda: A qualitative study. HIV/AIDS (Auckland, NZ). 2021;13:939.
13. Thompson TL, Haskard-Zolnierek K. Adherence and communication. Oxford Research Encyclopedia of Communication2016.
14. MacPherson P, Munthali C, Ferguson J, Armstrong A, Kranzer K, Ferrand RA, Ross DA. Service delivery interventions to improve adolescents' linkage, retention and adherence to antiretroviral therapy and HIV care. Tropical medicine & international health. 2015;20(8):1015-32.
15. Bervell B, Al-Samarraie H. A comparative review of mobile health and electronic health utilization in sub-Saharan African countries. Social Science & Medicine. 2019;232:1-16.
16. Free C, Phillips G, Galli L, Watson L, Felix L, Edwards P, et al. The effectiveness of mobile-health technology-based health behaviour change or disease management interventions for health care consumers: a systematic review. PLoS medicine. 2013;10(1):e1001362.
17. Creswell JW. A concise introduction to mixed methods research: SAGE publications; 2014.
18. Glasgow RE, Harden SM, Gaglio B, Rabin B, Smith ML, Porter GC, et al. RE-AIM planning and evaluation framework: adapting to new science and practice with a 20-year review. Frontiers in public health. 2019;7:64.
19. Graneheim UH, Lindgren B-M, Lundman B. Methodological challenges in qualitative content analysis: A discussion paper. Nurse education today. 2017;56:29-34.
20. Abiodun O, Ladi-Akinyemi B, Olu-Abiodun O, Sotunsa J, Bamidele F, Adepoju A, et al. A single-blind, parallel design RCT to assess the effectiveness of SMS reminders in improving ART adherence among adolescents living with HIV (STARTA trial). Journal of Adolescent Health. 2021;68(4):728-36.
21. Pop-Eleches C, Thirumurthy H, Habyarimana JP, Zivin JG, Goldstein MP, De Walque D, et al. Mobile phone technologies improve adherence to antiretroviral treatment in a resource-limited setting: a randomized controlled trial of text message reminders. AIDS (London, England). 2011;25(6):825.
22. Craig P, Dieppe P, Macintyre S, Michie S, Nazareth I, Petticrew M. Developing and evaluating complex interventions: the new Medical Research Council guidance. Bmj. 2008;337.
23. Mehra N, Tunje A, Hallström IK, Jerene D. Effectiveness of mobile phone text message reminder interventions to improve adherence to antiretroviral therapy among adolescents living with HIV: A systematic review and meta-analysis. Plos one. 2021;16(7):e0254890.

For peer review only

For peer review only - http://bmjopen.bmj.com/site/about/guidelines.xhtml

24. Tunje A, Jerene D, Kristensson Hallström I. Antiretroviral Therapy and Retention in Care Experiences and Needs of Adolescents Living with HIV in Southern Ethiopia. HIV/AIDS-Research and Palliative Care. 2021:999-1007.
25. McPherson AC, Gofine ML, Stinson J. Seeing is believing? A mixed-methods study exploring the quality and perceived trustworthiness of online information about chronic conditions aimed at children and young people. Health Communication. 2014;29(5):473-82.
26. Spinuzzi C. The methodology of participatory design. Technical communication. 2005;52(2):163-74.
27. Lindgren B-M, Lundman B, Graneheim UH. Abstraction and interpretation during the qualitative content analysis process. International journal of nursing studies. 2020;108:103632.
28. O'Connor C, Leyritana K, Doyle AM, Birdthistle I, Lewis JJ, Gill R, Salvaña EM. Delivering an mHealth adherence support intervention for patients with HIV: Mixed methods process evaluation of the Philippines connect for life study. JMIR Formative Research. 2022;6(8):e37163.
29. Sánchez SA, Ramay BM, Zook J, de Leon O, Peralta R, Juarez J, Cocohoba J. Toward improved adherence: a text message intervention in an human immunodeficiency virus pediatric clinic in Guatemala City. Medicine. 2021;100(10).
30. Mukund Bahadur K-C, Murrayb PJ. Cell phone short messaging service (SMS) for HIV/AIDS in South Africa: a literature review. MEDINFO 2010. 2010:530-4.
31. Lester R, Karanja S. Mobile phones: exceptional tools for HIV/AIDS, health, and crisis management. The Lancet infectious diseases. 2008;8(12):738-9.
32. Hlophe LD, Tamuzi JL, Shumba C, Nyasulu PS. Barriers to anti-retroviral therapy adherence among adolescents aged 10 to 19 years living with HIV in sub-Saharan Africa: A mixed-methods systematic review protocol. Plos one. 2022;17(9):e0273435.
33. Ammon N, Mason S, Corkery J. Factors impacting antiretroviral therapy adherence among human immunodeficiency virus–positive adolescents in Sub-Saharan Africa: a systematic review. Public health. 2018;157:20-31.
34. United Nations Social and Economic Council. Progress towards the Sustainable Development Goals. Report of the Secretary-General. NY. USA; 2022.
35. Creswell JW, Klassen AC, Plano Clark VL, Smith KC. Best practices for mixed methods research in the health sciences. Bethesda (Maryland): National Institutes of Health. 2011;2013:541-5.
36. Lincoln YS, Guba EG. Naturalistic inquiry: sage; 1985.
37. Schulz KF, Altman DG, Moher D. CONSORT 2010 statement: updated guidelines for reporting parallel group randomised trials. Journal of Pharmacology and pharmacotherapeutics. 2010;1(2):100-7.
38. Graneheim UH, Lundman B. Qualitative content analysis in nursing research: concepts, procedures and measures to achieve trustworthiness. Nurse education today. 2004;24(2):105-12.
39. World Medical Association. World Medical Association Declaration of Helsinki: ethical principles for medical research involving human subjects. Jama. 2013;310(20):2191-4.

Figure legend/caption

Figure 1 Flow Chart Diagram

Caption: The flowchart shows the flow diagram of mobile phone text messaging reminder in the intervention group.

For peer review only

For peer review only - http://bmjopen.bmj.com/site/about/guidelines.xhtml

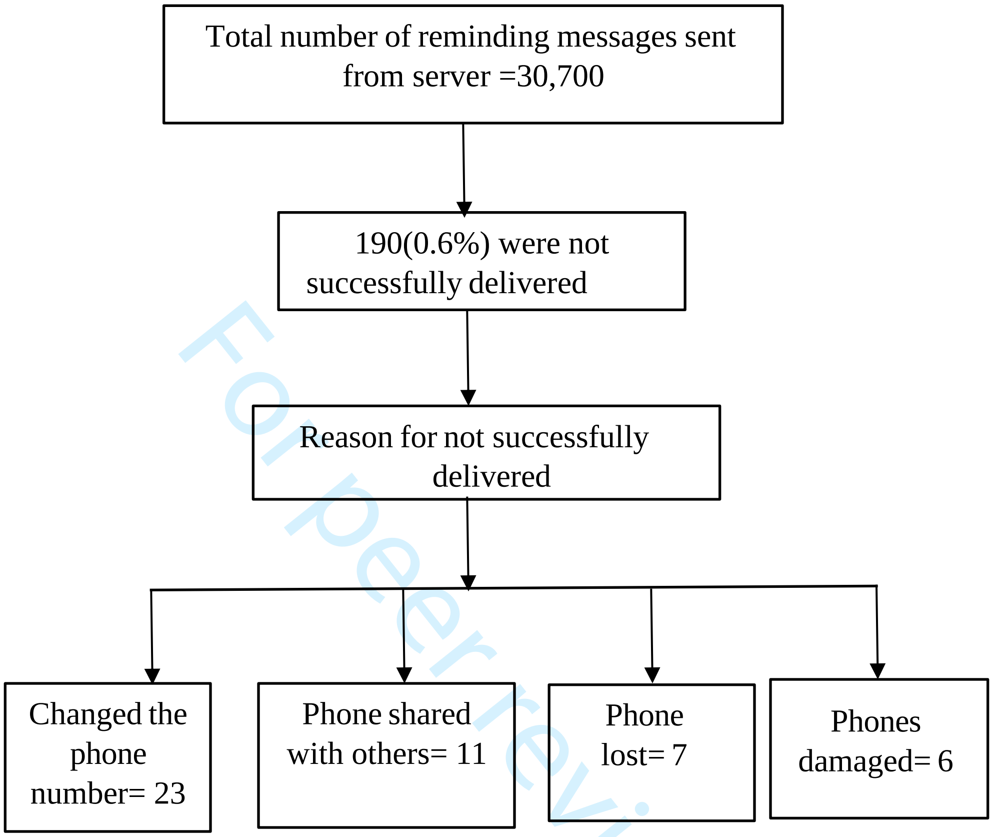

Figure 1. The flow diagram of mobile phone text messaging reminder in the intervention group

For peer review only

BMJ Open

Intervention fidelity and factors affecting the process of implementing a mobile phone text messaging intervention among adolescents living with HIV: a convergent mixed methods study in southern Ethiopia

Journal:	*BMJ Open*
Manuscript ID	bmjopen-2023-077128.R3
Article Type:	Original research
Date Submitted by the Author:	27-Apr-2024
Complete List of Authors:	Tunje, Abayneh; Lund University Faculty of Medicine, Department of Health Sciences; Arba Minch University, Public health<br>Persson, Helene ; Lund University Faculty of Medicine, Department of Health Sciences<br>Jerene, Degu; KNCV Tuberculosis Foundation<br>Hallstrom, Inger; Lunds Universitet,
**Primary Subject Heading**:	HIV/AIDS
Secondary Subject Heading:	Infectious diseases, Public health
Keywords:	Adolescent, HIV & AIDS < INFECTIOUS DISEASES, Information technology < BIOTECHNOLOGY & BIOINFORMATICS

SCHOLARONE™
Manuscripts

I, the Submitting Author has the right to grant and does grant on behalf of all authors of the Work (as defined in the below author licence), an exclusive licence and/or a non-exclusive licence for contributions from authors who are: i) UK Crown employees; ii) where BMJ has agreed a CC-BY licence shall apply, and/or iii) in accordance with the terms applicable for US Federal Government officers or employees acting as part of their official duties; on a worldwide, perpetual, irrevocable, royalty-free basis to BMJ Publishing Group Ltd ("BMJ") its licensees and where the relevant Journal is co-owned by BMJ to the co-owners of the Journal, to publish the Work in this journal and any other BMJ products and to exploit all rights, as set out in our licence.

The Submitting Author accepts and understands that any supply made under these terms is made by BMJ to the Submitting Author unless you are acting as an employee on behalf of your employer or a postgraduate student of an affiliated institution which is paying any applicable article publishing charge ("APC") for Open Access articles. Where the Submitting Author wishes to make the Work available on an Open Access basis (and intends to pay the relevant APC), the terms of reuse of such Open Access shall be governed by a Creative Commons licence – details of these licences and which Creative Commons licence will apply to this Work are set out in our licence referred to above.

Other than as permitted in any relevant BMJ Author's Self Archiving Policies, I confirm this Work has not been accepted for publication elsewhere, is not being considered for publication elsewhere and does not duplicate material already published. I confirm all authors consent to publication of this Work and authorise the granting of this licence.

For peer review only

Intervention fidelity and factors affecting the process of implementing a mobile phone text messaging intervention among adolescents living with HIV: a convergent mixed methods study in southern Ethiopia

Abayneh Tunje, PhD-student[1,2*] abayneh_tunje.tanga@med.lu.se, Helene Åvik Persson, PHN, PhD[1] helene.avik_persson@med.lu.se, Degu Jerene, MD, PhD[3] degu.dare@kncvtbc.org, Inger Kristensson Hallström, RN, PhD, Professor[1] Inger.kristensson_hallstrom@med.lu.se

Affiliations:

[1]Child and Family Health Unit, Department of Health Sciences, Faculty of Medicine, Lund University, Lund, Sweden.

[2] School of Public Health, College of Medicine and Health Sciences, Arba Minch University, Arba Minch, Ethiopia.

[3] KNCV Tuberculosis Foundation, The Hague, Netherlands.

***Corresponding author:**

Abayneh Tunje
abayneh_tunje.tanga@med.lu.se
The Faculty of Medicine
Department of Health Sciences
P.O. Box 157
221 00 Lund, Sweden

Abstract

Objective: To assess the intervention fidelity and explore contextual factors affecting the process of implementing a mobile phone text messaging intervention in improving adherence to and retention in care among adolescents living with HIV, their families, and their healthcare providers in southern Ethiopia.

For peer review only

Design: A convergent mixed-methods design guided by the process evaluation theoretical framework and the RE-AIM framework was used alongside a randomised controlled trial to examine the fidelity and explore the experiences of participants in the intervention.

Setting: Six hospitals and five health centres providing HIV treatment and care to adolescents in five zones in southern Ethiopia.

Participants: Adolescents (aged 10–19), their families and their healthcare providers.

Intervention: Mobile phone text messages daily for 6 months or standard care (control).

Results: 153 participants were enrolled in the process evaluation. Among the 153 enrolled in intervention arm, 78 (49.02%) were male, and 75 (43.8%) were female, respectively. The mean and standard deviation age of the participants is 15 (0.21). The overall experiences of implementing the text messages reminder intervention were described as helpful in terms of treatment support for adherence but had room for improvement. During the study, 30,700 text messages were sent, and fidelity was high, with 99.4% successfully delivered text messages during the intervention. Barriers such as failed text messages delivery, limitations in phone ownership, and technical limitations affected fidelity. Technical challenges can hinder maintenance, but a belief in the future of digital communication permeates the experiences of the text messages reminders.

Conclusions: Overall fidelity was high, and participants' overall experiences of mobile phone text messages were expressed as helpful. Contextual factors, such as local telecommunications networks and local electric power, as well as technical and individual factors must be considered when planning future interventions.

Trial registration: Pan African Clinical Trials **PACTR202107638293593, first registration** 20/07/2021.

Strengths and limitations with this study

For peer review only - http://bmjopen.bmj.com/site/about/guidelines.xhtml

- Secure and confidential delivery of high-volume messages through a 4-digit short code.
- The use of a mixed methods design and the inclusion of family members and healthcare providers is a strength of this process evaluation study.
- There was a limited number of adolescents´ family and healthcare providers included in the interviews.

INTRODUCTION

Globally, 38.4 million people were living with HIV in 2021. Of these, 1.71 million were adolescents aged 10–19, 88% of whom live in Sub-Saharan Africa (1, 2). To control the epidemic, the Joint United Nations Programme on HIV/AIDS (UNAIDS) launched the 95%–95%–95% targets aimed at HIV testing, treatment, and viral suppression by 2030 (3). However, by 2021, 75% percent of the 38.4 million people living with HIV (PLHIV) were receiving antiretroviral therapy (ART), a treatment gap of 5.9 million people (4). Moreover, achieving the recommended optimal adherence level of >95% which is required for ART to be effective, remains a challenge within the HIV care continuum (5). This poses a significant threat to HIV interventions in resource-limited settings (6).

Poor adherence to ART has several consequences, including increased risk of viral drug resistance and reduced treatment effectiveness toward viral suppression, leading to disease progression, greater risk of death, and increased risk of viral transmission (7). Some of the major barriers to ART adherence include socioeconomic status,(8, 9) fear of being stigmatised or discriminated against as a result of one's HIV status,(10) forgetting to take medication on time,(11) treatment fatigue,(12) and patient–provider communication (13). Several interventions to overcome these barriers have been studied in a variety of settings (14). The use of digital health strategies to enhance HIV treatment is one of the priority interventions in Sub-Saharan Africa (15). Targeted

For peer review only - http://bmjopen.bmj.com/site/about/guidelines.xhtml

digital client communication using mobile phone-based short text messaging has been recommended for adherence support for a range of health issues and adherence to antiretroviral treatment (16). The success and effectiveness of an mHealth intervention in achieving desired health outcomes is dependent on the context in which they are implemented (15, 16). Context plays a crucial role in either the success or failure of an intervention.

In addition to design and implementation, the success of interventions is diverse and may be influenced by factors such as sociodemographic and culture (17). Observing what is delivered in practice with close reference to the theory of the intervention can help evaluators in distinguishing the intervention fit in different contexts and changes that undermine intervention fidelity (15, 17). Trials should continue to rigorously assess outcomes, but also include integral process evaluations that use qualitative and quantitative data to develop and test hypotheses about how interventions work (18). In a multi-centre trial, a process evaluation is necessary to understand whether the intervention was implemented and received similarly across all sites (9). However, trials assessing mHealth adherence interventions for HIV often do not include process evaluations to examine the fidelity and quality of the intervention delivery, causal mechanisms for the health outcomes, contextual factors affecting the delivery, and costs of implementation (16, 19). Understanding the entire implementation process of an mHealth intervention allows practitioners to interpret the results and replicate the intervention in other contexts (20, 21). Therefore, this study aimed to evaluate the process of a mobile text messaging intervention conducted among adolescents living with HIV in Ethiopia.

METHODS

Study design

For peer review only - http://bmjopen.bmj.com/site/about/guidelines.xhtml

A convergent mixed-methods design (17) combining quantitative and qualitative techniques was used. The design was guided by the process evaluation theoretical framework (22) and structured by the RE-AIM dimensions (Reach, Effectiveness, Adoption, Implementation and Maintenance) (18) to evaluate intervention effectiveness alongside a randomised controlled trial (RCT) to examine the study's fidelity and to explore the experiences of the participants in the intervention.

The trial was registered in Pan African Clinical Trials Registry with the registration number PACTR202107638293593 on 17/07/2021.

Study setting and participants

Six hospitals and five health centres that provide HIV treatment and care to adolescents in the Gamo, Gofa, Konso, South Omo, and Wolayita zones in Southern Ethiopia were included in the main study. These zones are home to over 25 distinct ethnic groups, each with its own culture and context. Between July 5, 2022, and February 28, 2023, 306 adolescents living with HIV were included in the study, in either the intervention or in the control group. The quantitative data included process indicators, while the qualitative interviews included ten adolescents from the main trial, four adolescents' families, and two healthcare providers who were involved in the process evaluation. We used the following technique to identify eligible adolescents: first, we identified the number of adolescents of eligible age in each facility. Then, using their address, we called each adolescent and their parents or caregivers directly via phone numbers, care providers, or adherence supports. Then, data collectors approached adolescents and posed literacy questions about their ability to read text by displaying sample texts. Then, eligible participants in the study were asked to give consent and assent. Thus, adolescents aged 10 to 19 years, diagnosed with HIV, currently participating in ART care, and planning to stay in the designated facility for at least six months following study enrolment were eligible. Participants who were above the age limit, did not

For peer review only - http://bmjopen.bmj.com/site/about/guidelines.xhtml

disclose their HIV status, were unable to read text, or had a disability were all excluded. Finally, the data collector showed each eligible adolescent how to use their mobile device, including text saving and deletion.

Theoretical Framework of Process Evaluation

The RE-AIM framework (18) was used for the process evaluation to comprehensively examine the level of implementation. This involves understanding the implementation of interventions in terms of (1) fidelity, defined as to what extent the intervention was implemented consistently with the underlying theory as planned; (2) delivery, defined as to what extent all of the intended activities, training, and materials were provided to program participants; (3) reach, defined as the absolute number, proportion, and representativeness of individuals who are willing to participate in a given initiative, intervention, or program; (4) receipt, defined as how participants reacted to specific aspects of the intervention; and (5) context, defined as what contextual factors influence implementation or the intervention outcome.

Description of the intervention

The MRC framework,(22) a systematic literature review,(23) interviews with adolescents,(24) and the theoretical health belief model (HBM) (25) were used to develop the intervention. Adolescents living with HIV, healthcare providers, and research project technical advisory team members participated in the design (26) under the guidance of a research advisory committee. The HBM is a theoretical model that has been constructed from six domains: susceptibility, severity, barriers, benefit, cues to action, and self-efficacy. After the intervention's development, text messages were uploaded to the server, and an automated message-pushing software was used to send tailored daily text messages using a four-digit code from Ethiopia Telecom. This code helps adolescents in

For peer review only - http://bmjopen.bmj.com/site/about/guidelines.xhtml

avoiding messaging source-related stigma and distinguishing intervention messages from other messages.

Other various configurations such as wide-area network (WAN) internet protocol (IP), local area network (LAN) IP, Ethio telecom central server IP, a virtual private network (VPN) line, and office servers were used as infrastructure, as required to support the study. The text messages were categorised and delivered based on each adolescent's customised medication schedule and time. The software sent the message 15 minutes before each participant's medication time. The type of message was short and mainly focused on advice about the benefits of taking on time, the risk/consequences of missing/not taking on time, encouragement, and without mentioning anything about HIV or the name of the drug. For example, "Those who use it properly know its benefits", "I'm taking it properly and on time". For example, these messages in Amharic are "በትክክል የሚጠቀሙት ጥቅሞቹን ያውቃሉ) and በትክክል እና በሰዓት እወስዳለሁ respectively. The server was checked daily to troubleshoot network/cell phone issues. Based on the server information, the first author contacted the facility level care provider to contact the adolescent with corrective actions; for example, subscriber identity module card (SIM card) replacement, damaged mobile phones, and network troubleshooting for maintenance and replacement.

Sample and data collection

The quantitative data was collected from the server, activity logs, an adverse event monitoring form, and an interview with adolescents. The researcher collects data on intervention fidelity by tracking the number of text messages sent, delivered, and failed from the server daily.

Furthermore, each facility representative documented any adverse event related to the trial, filled the form, and sent it to researchers every week. Furthermore, during the follow-up data collection phase, adolescents asked if they received text messages on a regular schedule.

For peer review only - http://bmjopen.bmj.com/site/about/guidelines.xhtml

A purposive sampling of 10 adolescents with HIV (aged 12–19), four family members/relatives (aged 36–45), and two healthcare providers (aged 31–36) from five different healthcare facilities were asked to be interviewed about their experiences in the intervention between July 5, 2022, and February 28, 2023, at healthcare facilities by the first author. The interviews were conducted in all respondent languages, such as Amharic, Wolaytigna, Gamogna, Gofigna, Arigna, and Konsegna in a quiet and confidential area at the respective health facilities by the first author and one research assistant and lasted on average 30 minutes. All interviews were audio-recorded with the permission of the participants.

An interview guide was used to gain a rich, in-depth understanding, and to allow the participants to concretise their experiences of their use of text messages, communication, and technical aspects of the intervention. The interview guide included subsections adapted to the participants, i.e., adolescents, families, and healthcare providers (interview guide attached as supplement file). Hence, the questions were different, depending on to whom they were addressed. The introductory question to the adolescents was: "Are you receiving the mobile text-message reminder from the hospital?", followed by follow-up questions such as, "What do you think about the messages?" The families were initially asked "How do family/social related factors affect ART adherence among adolescents?" Healthcare providers were asked "How did you experience communication with the adolescents with regard to the intervention?" Follow-up questions and prompts were used throughout the whole interview.

The interviews were audiotaped, transcribed verbatim, and translated from Amharic to English by the first author, and checked for accuracy by one independent language expert.

Data analysis

For peer review only - http://bmjopen.bmj.com/site/about/guidelines.xhtml

The number of SMS messages sent and delivered to participants were tracked as a measure of intervention fidelity. When SMS messages did not arrive on the phone, the project advisory team investigated the external network and other infrastructure issues. If the message could not be delivered during this period, the computer automatically recorded it as a delivery failure and sent the message again. Data captured on the delivery status of the SMS messages were recorded as: delivered (the phone had reception marked as green); undelivered (the phone was switched off or marked as red in the server). The program could not record whether messages delivered to the phones were opened.

The descriptive statistical analysis was used to summarize data in frequency, percentages, mean and standard deviation. The quantitative effectiveness data is analyzed separately and not included in the process evaluation. The RE-AIM outcomes framework components of Reach, Effectiveness, Adoption, Implementation, and Maintenance were used to present mixed method findings about the delivery of a reminder message, success, and intervention barriers for the intervention implementation process (18).

Interviews were analysed by using the principles for content analysis described by Graneheim and Lundman (19, 27). First, the transcripts were read several times to gain a sense of the whole by the first and the last author. Thereafter, meaning units were identified and condensed to reduce the text while maintaining the core meaning. The first author developed a coding frame for the analysis by using NVivo software, which were discussed by all authors. Condensed meaning units were then labelled with a code, which were kept close to the text on a manifest level. Next, sub-themes were created from the code frame by the first and last authors. The sub-themes were then abstracted into themes. Finally, the underlying meaning—the latent content—was described into a main theme. Thereafter one of the authors (HÅP) read the transcripts, extracted meaning units, codes, and

For peer review only - http://bmjopen.bmj.com/site/about/guidelines.xhtml

themes. The content was discussed and reflected upon together with all authors and lasted until an agreement was reached.

Patient and Public Involvement

Representatives of adolescents were involved in the development of messages for the SMS reminders and interview guides. Their feedback was used to adapt its contents and to set strategies to promote inclusivity in study participation during participant recruitment. In addition, participants, healthcare providers, and the participants' families were involved in reporting any incidence related to mobile devices and other issues.

RESULTS

The characteristics of adolescents assigned in the intervention arm are shown in table 1.

Table 1. Participant characteristics

**Characteristics of adolescents assigned in the intervention**		
**Characteristics**		Frequency /Mean (SD)/ Percent
Adolescent age, Mean (standard deviation (SD))		15(0.21) years
Adolescents age category	10-14	60 (39.22)
	15-19	93 (60.78)
Adolescents gender	Male	78 (49.02)
	Female	75 (43.8)
Languages of adolescent during interview	Amharic	62 (40.52)
	Wolaytigna	27 (17.65)
	Gamogna	39 (25.49)
	Gofigna	13 (8.5)
	Arigna	6 (3.3)
	Konsegna	6 (3.3)
Educational status of adolescent	Grade 1-8	97 (63.40)
	Grade 9 and above	56 (36.60)
Residence of adolescent	Urban	111(72.55)
	rural	42 (27.45)
Living arrangement	Live alone	10 (6.54)
	Live with family	143 (93.46)
Psycho-social peer-support	Participate	47 (30.72)

For peer review only - http://bmjopen.bmj.com/site/about/guidelines.xhtml

	Do not Participate	106 (69.28)
Interviewee characteristics		
Adolescents (Sex/age)	Male (10-14 years)	2
	Female (10-14 years)	3
	Male (15-19 years)	2
	Female (15-19 years)	3
Adolescents level of education	Grade 1-8	5
	Grade 9 and above	5
healthcare providers (Sex)	Male	1
	Female	1
Parent/care giver (Sex)	Non-biological care giver	2 (1 male and 1 female)
	Biological	2 (1 mother and 1 father)

The results are structured using the RE-AIM framework (18) and corresponding themes (Table 2).

Table 2. Overview of the qualitative themes related to the dimensions in the RE-AIM framework.

**RE-AIM dimension (18)**	**Definition (18)**	**Qualitative theme**
Reach	The number, proportion, and representativeness of eligible individuals who participate in each initiative.	
Effectiveness	The impact of an intervention on the relevant outcomes, including potential adverse effects, quality of life, and economic outcomes.	Text message reminders were helpful for adherence but there was room for improvement
		Text messages reminders support adherence
		Feeling comfortable receiving text messages reminders
		The code delivered with text messages reminders minimises the risk of stigma.
Adoption	The reach and effectiveness/efficacy of an intervention at the setting level.	Supporting the text messages reminders
Implementation	The intervention agents' fidelity to the various elements of an intervention's protocol, including consistency of intended delivery.	Failing text messages delivery influences medication intake
		Limitations in phone ownership

For peer review only - http://bmjopen.bmj.com/site/about/guidelines.xhtml

		Technical limitations
Maintenance	Connects with both setting-level indicators (the extent to which a program or a policy becomes part of organisational practices and policies), and individual-level indicators (monitoring of effectiveness of an intervention or program six months or more after the most recent contact).	Seeing potential in SMS services
		Possibilities of text messages reminders

219 **Reach:**

220 The total number of individuals that were assessed for eligibility was 435, of which 306 (70.34 %)
221 meet eligibility criteria. The remaining 129 participants excluded for the following reasons: 27
222 (20.9%) were unable to stay in the study area for the duration of the study, 39 (30.2%) were unable
223 to read, 24 (18.6%) were did not know their status, 21 (16.3%) were could not reached, 13 (10.1%)
224 refused to participate, and 5 (3.9%) had an impairment. Thus, 306 participants were enrolled and
225 randomised: n =153 in the intervention, and n =153 in the control group. Among the 153 enrolled
226 in intervention arm, 78 (49.02%) were male, and 75 (43.8%) were female, respectively. The mean
227 and standard deviation age of the participants is 15 (0.21). Of these, 111 (72.55%) resided in an
228 urban area, 97 (63.40%) had primary school level education, and 10(6.54%) live alone(orphan).

229 **Effectiveness:**

230 *The overall rate of retention at six months in the intervention group was 152/153 (99.4%), while*
231 *in the control group was 151/153 (98.69%).*

232 *Text messages reminders were helpful for adherence but there was room for improvement.*

233 The overall experiences of the intervention among adolescents living with HIV was that text
234 messages reminders were helpful when it came to their adherence to medication, but direct access

For peer review only - http://bmjopen.bmj.com/site/about/guidelines.xhtml

to phones and better timing of messages should be assured to increase their functioning and usability. The adherence was improved, and was the best achieved since the intervention was introduced. After six months of follow-up, the intervention group had a higher rate (114/152, 75%) than the control group (77/151, 51%). Despite this, more effort was requested concerning technical issues to make the reminders even more helpful. The participants described facilitating aspects like increased medication intake and communication. Also, the well-being of the adolescents and the support function was highlighted. However, technical problems like lost telephones or text messages reminders arriving after medication time were emphasised as barriers.

Text messages reminders support adherence

They described that before the intervention, their medication was often forgotten, which resulted in it being taken later than it should have been due to forgetfulness. Sometimes the adolescent realised that they had forgotten to take their medication but then waited until dinner to take it. The adolescents described that text messages reminders were associated with taking their medicine and delays were avoided. Also, family members described situations when the adolescent's medication routines were interrupted, and where text messages reminders played a big role.

> *"…Yes, it supports treatment adherence, because most of time we follow the television program but sometimes the power goes off, we missed the exact time to see medication time. Currently this text message comes 10 to 15 minutes before our medication time."*
>
> *(Father, aged 45)*

Feeling positive about the text messages reminder

For peer review only

The text messages reminders contributed to positive attitudes and to decreased stigmatisation for adolescents, even when HIV/AIDS stigma is deeply rooted in the culture. Thus, the perception of the adolescents was that the text messages reminders played an important role for their overall well-being, because sometimes they had low moods. The text messages reminders were described as offering hope and supported the adolescents in achieving consistency in their medication adherence which helped them avoid, for example, fatigue.

"…The benefit of this message is it comes always and reminds me to take the drug on time. The other benefit is when I receive the message, I feel good." (Adolescent, aged 13)

The code-delivered text messages reminders minimise the risk of stigma

The adolescent felt safe regarding privacy in the text messages reminders due to a code sent out to their telephone. The healthcare provider noticed a change in the views of both adolescents and family regarding increased trust and improved engagement with healthcare facilities during the intervention. Family expressed receiving coded text messages reminders could enable adolescents to live like other HIV-free adolescents. However, the adolescents indicated that after their family's HIV status was made public, they were subjected to stigma, which was reflected in the interview. Some members of the families were open with their HIV disclosure while others were not.

"My status has already been made public, and everybody knows about my family as well ... However, my husband lives in another area because of work, and knows his HIV status and makes things secret. He did not even take his medication when he come back home." (Mother, aged 45)

Adoption:

In this study, eleven health facilities with 153 participants received an intervention across 5 different geographic areas. According to healthcare providers, the rate of retention among

For peer review only - http://bmjopen.bmj.com/site/about/guidelines.xhtml

adolescents has increased since the study began in the hospital, as described in the qualitative findings under a sub-theme.

".... By the way, in terms of retention, we have observed the best retention rate ever in this year since ART started in this hospital. Of course, our follow-up is there and there are other partners like CDC and ICAP. But this messaging has augmented the service we provide, and it increased our communication with them and helped them remind their medication irrespective of inconsistencies in messaging and medication time; the message delivery reminds them something targeted." (healthcare provider, aged 36)

Supporting the text messages reminders

The healthcare providers emphasised that text messages reminders were an important support for adolescents in their medication intake to avoid poor adherence. In addition to this, families indicated that they had a mission to support the adolescents in avoiding ART treatment fatigue. Several adolescents expressed that they had received support from their family concerning medication intake during the intervention. Thus, the text messages reminders were perceived as helpful, as they made participants feel happy and hopeful, even if they felt angered or were in a depressed mood that day.

"…The messages may help people pass good days, despite days that do not carry good fortune. It therefore helps people have good days and reminds them of medication times. One day, for example, being consumed with some ideas, I did not recall that I have medication to take; it is this text message that triggered me take my medicine." (Adolescent, aged 19)

Implementation:

There were 306 mobile phones provided to adolescents during assignments into intervention and control groups. The advisory committee was involved in the implementation of the intervention

For peer review only - http://bmjopen.bmj.com/site/about/guidelines.xhtml

using standard operational procedure (SOP). During the study, 30,700 SMS text messages were sent to 153 study participants in the intervention group (Figure 1).

Overall, intervention fidelity was high with (30,510/30,700) or 99.38% successfully delivered SMS text messages during the intervention.

The non-receiving participants in the intervention group were tracked and cross-checked using individual participant codes from the server, and then approached by each health facility to confirm whether they were to be followed-up.

Changing phone numbers/SIM cards, phones being damaged, lost, or shared with others were identified as participant-related reasons for not receiving the messages, and were registered in the logbooks, and corrective measures were used either to replace phones with new devices or repair/maintain the phones as soon as was feasible.

Failing text message delivery influences medication intake

The adolescents identified several reasons for not receiving messages. In connection with the technical aspect, the adolescents highlighted problems such as not having a charged phone battery or having lost their phone. During the feasibility study period, there was fluctuation in messaging time, and the text message reminders arrived after their medication time. Another issue observed by both healthcare providers and family members was a missing SIM card. Furthermore, the continuity in the text messages reminders was requested by the adolescents to continue the benefits.

> *"… the receiving time varies; sometimes I receive it 10 minutes before medication time, which is 3p.m. local time in the evening, and sometimes it arrives after I take the drug." (Adolescent, aged 13)*

Limitations in phone ownership

For peer review only - http://bmjopen.bmj.com/site/about/guidelines.xhtml

Sometimes adolescents were not permitted to use the telephone by themselves, due to restrictions from their family, as they thought that the adolescents were too young to use the telephone. Instead, the family used the telephone, which resulted in text messages reminders never reaching the adolescent. The absence of SIM cards may be because adolescents did not receive a SIM card, or because someone else in the family was using them. The family reported that they passed information from healthcare providers to the adolescents. Some of the families lacked information about the intervention and did not know what to do when the telephone arrived. The healthcare providers emphasised that some families denied the adolescents use of the telephone, which could cause problems, and text messages reminders were not received. Furthermore, the importance of informing families about the intervention was stressed, as several adolescents were restricted from using the telephone.

"…I think families should be communicated with regarding why their children are provided with mobile phones from the hospital, so as to avoid denial to access or adults using the phones for themselves." (Healthcare provider, aged 36)

Technical limitations

One major issue stressed by adolescents, families, and healthcare providers, was that the electric power system went off quite often in their communities, which caused problems with receiving text messages reminders if the battery in the telephone was not charged. The adolescents usually did not experience problems with delays in text messages reminders, but they had observed that the text messages reminders did not always arrive on time. The messages could come long before their scheduled medication time or sometimes very close to or after their scheduled medication time. Sometimes the text message reminder was absent for entire days.

For peer review only - http://bmjopen.bmj.com/site/about/guidelines.xhtml

"...The message helped me a lot to remember my medication time. However, the skips in message delivery dates and untimely arrivals should be corrected so that it is more helpful." (Adolescent, aged 18)

Maintenance:

After initial configuration, a single server can send text messages to thousands of participants across a large geographic area. In this study, a server sent text messages to 153 participants across five different geographic areas. The technical challenges experienced in delivering the intervention were related to navigating the messaging server and feedback responses from the participants. The non-receiving participants in the intervention group were tracked and cross-checked using a four-digit individual participant codes from the server and were then approached by each facility to confirm whether they were indeed in that group or lost. Despite technical barriers, and adverse conditions, and a belief in the future of text messages reminders emerged, which was also expressed in the qualitative interviews.

Seeing potential in SMS services

The benefits of SMS reminders were perceived as valuable; especially when adolescents missed appointments for medication refills and virus load tests. Before the intervention, there were difficulties getting in touch with the adolescents, because they lived in rural areas and did not have a telephone. The healthcare providers thought about how to incorporate text messages reminders into routine health care. A wish among the adolescents was expressed that more text messages reminders could be helpful in raising awareness about their condition. Both the adolescents and the healthcare providers raised a need to continue the text message reminders.

"I think this must continue. The mobile provision has played significant roles in adherence and retention, as many of the adolescents do not have phones…because of this provision, adolescents can be reached directly with health messages and receive important lessons just from their phones." (Adolescent, aged 19)

For peer review only - http://bmjopen.bmj.com/site/about/guidelines.xhtml

Possibilities with text messages reminders

The healthcare provider described that possibilities to communicate with the adolescents had increased after the intervention, and the adolescents' attitudes towards healthcare facilities had become more positive. The adolescents found the improved digital communication to be an advantage because the healthcare provider no longer needed to call their family but could call them directly.

> *"Previously, healthcare providers from hospital reached me through neighbourhood phone numbers. She now uses this number to reach me directly through the phone provided by the hospital. This is a benefit for my privacy and for the information we discuss over the phone." (Adolescent, aged 18)*

DISCUSSION

Our process evaluation which was based on the RE-AIM framework (18) showed that intervention fidelity was high with only about 0.6% of the SMS messages not delivered as intended. Both providers and patients described the SMS reminders as being helpful. A few barriers were noted including glitches at telecommunication networks, electric power disruptions, issues with phone ownership at household levels. Our results highlight that SMS message reminders can be delivered with high coverage if proper processes are followed. The 4-digit short code messaging system was a key enabler in ensuring confidentiality and security of the messages. Further scale-up of such interventions will depend on addressing broader systems issues including phone ownership, fixing network interruptions and reliable power sources. However, a study conducted in Philippines showed that intervention fidelity was low (77.9%) in the message group. The identified reason for low intervention fidelity was poor reliability of local telecommunication networks, and frequency of messages received (28). The local context for cellular phone infrastructure and operational challenges, such as multiple users on a single cell phone, has an impact on text messaging

For peer review only - http://bmjopen.bmj.com/site/about/guidelines.xhtml

interventions (29). It is important to assess the setting before using the SMS intervention as a strategy to improve adherence. There is emerging evidence that mobile phones can play an important role in healthcare delivery, especially in resource-limited settings (30). SMS text messaging is a particularly useful application that can be used to collect or share information and to enhance communication between healthcare personnel and patients in a low-cost manner (31). Treatment support was highlighted as an important factor for adolescents to avoid poor adherence. Text messages could be a facilitator for adolescents to remain hopeful and avoid unwanted side effects given that previous research (32, 33) has shown that a lack of support systems was perceived to negatively affect adherence among young people. Our findings provide additional evidence in support of research to identify effective interventions to improve adherence and retention in care for adolescents with HIV and enhance their engagement in these services. Thus, findings of this process evaluation can guide further research towards interventions concerning readiness among healthcare facilities, because settings can impact implementation. Giving importance to the voice of adolescents living with HIV can offer hope to many more in the same situation. This study thereby has a potential clinical impact and contributes to UN Sustainability Goal (34).

Methodological concerns/ limitations

There are both strengths and limitations identified in this process evaluation. First, the intervention has several strengths, and a key strength is the use of a 4-digit short code for text messages that enables rapid, high-volume outbound messaging, is easy to read and remember, and assists text recipients in identifying the sender based on their preferences for message delivery time, message content, and messaging language. The use of the unique code can contribute to maintaining security and confidentiality among participants.

For peer review only - http://bmjopen.bmj.com/site/about/guidelines.xhtml

Secondly, the messaging server is adaptable and user-friendly, as the researcher or local IT provider can add and remove new data fields to navigate dropped or missed individuals and update SMS text message content to avoid message-related fatigue.

In addition, the process evaluation also has several strengths that were identified. The first strength is the use of mixed methods, where different forms of data—both quantitative and qualitative methods—are combined to capture the multi-dimensionality of the intervention. The data supplement one another and aid in obtaining a comprehensive picture of the study results, which can contribute to a deeper understanding of the participants' experiences (35). If the participants are given the opportunity to highlight their own individual priorities, it might be possible for us to improve our understanding of the impact of the intervention.

Finally, three different perspectives (adolescents, families, and healthcare providers) were included in the qualitative interviews, which allowed different voices to be heard. However, limited numbers of included participants from adolescents' families and healthcare providers can limit the study's transferability (36). The way research results are presented can be influenced by their nature and outcome, leading to what is known as reporting bias. To minimize outcome reporting bias, the study protocol was pre-registered in the pan-African clinical trials registry. A composite adherence measurement was used, and CONSORT recommendations were followed (37). The fact that adherence data was self-reported by healthcare professionals within the same facilities through interviews may have a significant impact on the study's validity and make it prone to social desirability bias. However, in this study, the primary author conducted the interviews in a separate, private room. Furthermore, providing cell phones to adolescents during recruitment may reduce the likelihood of interview refusal in the intervention phase, causing acquiescence bias.

For peer review only - http://bmjopen.bmj.com/site/about/guidelines.xhtml

Trustworthiness is the most commonly used criterion for evaluating qualitative content analysis, and it is often expressed using terms such as credibility, conformability, dependability, and transferability (36). To ensure the credibility of the study, an appropriate data collection method needs to be used(38). This study used separate interview questions for adolescents, family, and professional interviews to explore their experiences and issues with mobile text messaging interventions. In this study, the five-dimension components of the RE-AIM framework were used to collect information about the intervention setting, implementation, personnel and circumstances, and findings by considering individual level and external context challenges(18). To ensure the dependability of the study, an interview guide adapted to the participants, and audio-recorded was used. For the confirmability of this study, the first author transcribed the audio data to verbatim text and translated from Amharic to English by considering both manifest and latent content. Then, all authors involved in reading the transcripts, extracted meaning units, codes, and data analysis.

Finally, the authors previous qualitative research experiences and knowledge, as well as a bracketing technique were used to increase the trustworthiness of their findings. Accordingly, the primary author maintained a positive relationship with participants during intervention follow-ups and established independence from caregiving facilities to minimize social desirability bias and boss views. To reduce bias and subjective interpretation, interviews were audio recorded, transcribed, and thoroughly evaluated, with participant quotes used to support the authors' findings.

Conclusion

The mobile phone text messaging intervention to support ART adherence and retention in care was well-received by participants, and overall intervention fidelity was high. However, the intervention's feasibility was dependent on the reliability of local telecommunication networks, local electric power, and monitoring of adolescents' families, all of which had a significant impact

For peer review only - http://bmjopen.bmj.com/site/about/guidelines.xhtml

on the intervention's usability, fidelity, and doses received. The participants experienced the text messaging function as helpful, therefore the development of different aspects, such as addressing technical problems, would be desirable to further improve impact. Further research needs to be directed toward readiness within the healthcare facilities to capture an increased understanding of barriers and facilitators in implementation.

Author affiliations

[1]Child and Family Health Unit, Department of Health Sciences, Faculty of Medicine, Lund University, Lund, Sweden.

[2] School of Public Health, College of Medicine and Health Sciences, Arba Minch University, Arba Minch, Ethiopia.

[3] KNCV Tuberculosis Foundation, The Hague, Netherlands.

Acknowledgements

The authors sincerely thank the participants for sharing their experiences, and all staff working in the ART clinics for facilitating the study.

Contributors

AT, DJ, and IKH conceptualized and developed the study design. AT collected the data and transcribed verbatim and translated from Amharic to English. DJ and IKH supervised data collection and study implementation. All authors read the transcripts, extracted meaning units, codes, and data analysis. Sub-themes were created from the code frame by the first and last authors. AT and HÅP drafted the manuscript, and all authors contributed to critical revision and approved the final manuscript.

Funding

For peer review only - http://bmjopen.bmj.com/site/about/guidelines.xhtml

The project was externally funded by FORTE: Swedish Research Council for Health, Working Life and Welfare Life (FORTE) (https://forte.se/en/) program support 2018–01399 and the Swedish Research Council(https://www.vr.se/english.html) program support VR 2016-05706. The study protocol has undergone a peer review by the funding body. The funders had no role in study design, writing the study protocol, the decision to publish the study protocol, or preparation of the manuscript.

Competing interests

None declared.

Patient and public involvement

Patient consent for publication

Not applicable.

Ethics approval

The study was conducted according to the guidelines in the Declaration of Helsinki (39) and approved by the Swedish Regional Ethical Review Board (Dnr 2019-03433), National Research Ethics Review Committee in Ethiopia (MoSHE/RD/142/2869/20), and the Institutional Research Ethics Review Board in Arba Minch University (IRB-113/11). The participants received both oral and written information about the purpose of the study, about the confidential treatment of the data and the voluntary nature of participation. Written Informed consent was provided from all participants over the age of 18 before the study began. Similarly, parental written consent and adolescent written assent were obtained from all participants under the age of 18 before the start of the trial.

For peer review only

For peer review only - http://bmjopen.bmj.com/site/about/guidelines.xhtml

Provenance and peer review

Not commissioned, externally peer reviewed.

Data availability statement

Extra data can be accessed via the Dryad data repository at https://doi.org/10.5061/dryad.n8pk0p31s

Supplemental material

Semi-structured interview guide was attached **Open access**

This is an open access article distributed in accordance with the Creative Commons Attribution Non-Commercial (CC BY-NC 4.0) license, which permits others to distribute, remix, adapt, build upon this work non-commercially, and license their derivative works on different terms, provided the original work is properly cited, appropriate credit is given, any changes made indicated, and the use is non-commercial. See: http://creativecommons. org/licenses/ by-nc/ 4.0/

ORCID

Abayneh Tunje (orcid.org/0000-0002-0957-0216)

Degu Jerene (orcid.org/ 0000-0001-7853-4211)

Helene Åvik Persson (orcid.org/0000-0002-7862-5444)

Inger Kristensson Hallström (orcid.org/0000-0002-9402-6230)

References

1. UNAIDS. Global HIV & AIDS statistics — Fact sheet: https://www.unaids.org/en/resources/fact-sheet. 2021.
2. UNICEF. Global and regional trends. 2022 [Available from: https://data.unicef.org/topic/hivaids/global-regional-trends/.
3. Duncombe C, Ravishankar S, Zuniga JM. Fast-Track Cities: striving to end urban HIV epidemics by 2030. Current Opinion in HIV and AIDS. 2019;14(6):503-8.
4. World Health Organization. HIV data and statistics. World Health Organization (WHO) Available online: https://www who int/teams/global-hiv-hepatitis-and-stis-programmes/hiv/strategic-information/hiv-data-and-statistics. 2021.

For peer review only

For peer review only - http://bmjopen.bmj.com/site/about/guidelines.xhtml

5. African Network for the Care of Children Affected by HIV/AIDS – ANECCA. Handbook on Pædiatric AIDS in Africa by the African Network for the Care of Children Affected by AIDS 2017. Available from: https://www.anecca.org/wp-content/uploads/2018/12/ANECCA-HANDBOOK-2017-ENGLISH-VERSION_20170530_web.pdf.

6. Pugh LE, Roberts JS, Viswasam N, Hahn E, Ryan S, Turpin G, et al. SYSTEMATIC REVIEW OF INTERVENTIONS AIMED AT IMPROVING HIV ADHERENCE TO CARE IN LOW-AND MIDDLE-INCOME COUNTRIES. Journal of Infection and Public Health. 2022.

7. Sefah IA, Mensah F, Kurdi A, Godman B. Barriers and facilitators of adherence to antiretroviral treatment at a public health facility in Ghana: a mixed method study. Hospital Practice. 2022;50(2):110-7.

8. Hudelson C, Cluver L. Factors associated with adherence to antiretroviral therapy among adolescents living with HIV/AIDS in low-and middle-income countries: a systematic review. AIDS care. 2015;27(7):805-16.

9. Rudy BJ, Murphy DA, Harris DR, Muenz L, Ellen J. Patient-related risks for nonadherence to antiretroviral therapy among HIV-infected youth in the United States: a study of prevalence and interactions. AIDS patient care and STDs. 2009;23(3):185-94.

10. Gill MM, Ndimbii JN, Otieno-Masaba R, Ouma M, Jabuto S, Ochanda B. Adherence challenges and opportunities for optimizing care through enhanced adherence counseling for adolescents with suspected HIV treatment failure in Kenya. BMC Health Services Research. 2022;22(1):1-11.

11. MacDonell K, Naar-King S, Huszti H, Belzer M. Barriers to medication adherence in behaviorally and perinatally infected youth living with HIV. AIDS and Behavior. 2013;17(1):86-93.

12. Ajuna N, Tumusiime B, Amanya J, Awori S, Rukundo GZ, Asiimwe JB. Social networks and barriers to ART adherence among young adults (18–24 years) living with HIV at selected primary health facilities of South-Western Uganda: A qualitative study. HIV/AIDS (Auckland, NZ). 2021;13:939.

13. Thompson TL, Haskard-Zolnierek K. Adherence and communication. Oxford Research Encyclopedia of Communication2016.

14. MacPherson P, Munthali C, Ferguson J, Armstrong A, Kranzer K, Ferrand RA, Ross DA. Service delivery interventions to improve adolescents' linkage, retention and adherence to antiretroviral therapy and HIV care. Tropical medicine & international health. 2015;20(8):1015-32.

15. Bervell B, Al-Samarraie H. A comparative review of mobile health and electronic health utilization in sub-Saharan African countries. Social Science & Medicine. 2019;232:1-16.

16. Free C, Phillips G, Galli L, Watson L, Felix L, Edwards P, et al. The effectiveness of mobile-health technology-based health behaviour change or disease management interventions for health care consumers: a systematic review. PLoS medicine. 2013;10(1):e1001362.

17. Creswell JW. A concise introduction to mixed methods research: SAGE publications; 2014.

18. Glasgow RE, Harden SM, Gaglio B, Rabin B, Smith ML, Porter GC, et al. RE-AIM planning and evaluation framework: adapting to new science and practice with a 20-year review. Frontiers in public health. 2019;7:64.

19. Graneheim UH, Lindgren B-M, Lundman B. Methodological challenges in qualitative content analysis: A discussion paper. Nurse education today. 2017;56:29-34.

20. Abiodun O, Ladi-Akinyemi B, Olu-Abiodun O, Sotunsa J, Bamidele F, Adepoju A, et al. A single-blind, parallel design RCT to assess the effectiveness of SMS reminders in improving ART adherence among adolescents living with HIV (STARTA trial). Journal of Adolescent Health. 2021;68(4):728-36.

21. Pop-Eleches C, Thirumurthy H, Habyarimana JP, Zivin JG, Goldstein MP, De Walque D, et al. Mobile phone technologies improve adherence to antiretroviral treatment in a resource-limited setting: a randomized controlled trial of text message reminders. AIDS (London, England). 2011;25(6):825.

22. Craig P, Dieppe P, Macintyre S, Michie S, Nazareth I, Petticrew M. Developing and evaluating complex interventions: the new Medical Research Council guidance. Bmj. 2008;337.

For peer review only - http://bmjopen.bmj.com/site/about/guidelines.xhtml

23. Mehra N, Tunje A, Hallström IK, Jerene D. Effectiveness of mobile phone text message reminder interventions to improve adherence to antiretroviral therapy among adolescents living with HIV: A systematic review and meta-analysis. Plos one. 2021;16(7):e0254890.
24. Tunje A, Jerene D, Kristensson Hallström I. Antiretroviral Therapy and Retention in Care Experiences and Needs of Adolescents Living with HIV in Southern Ethiopia. HIV/AIDS-Research and Palliative Care. 2021:999-1007.
25. McPherson AC, Gofine ML, Stinson J. Seeing is believing? A mixed-methods study exploring the quality and perceived trustworthiness of online information about chronic conditions aimed at children and young people. Health Communication. 2014;29(5):473-82.
26. Spinuzzi C. The methodology of participatory design. Technical communication. 2005;52(2):163-74.
27. Lindgren B-M, Lundman B, Graneheim UH. Abstraction and interpretation during the qualitative content analysis process. International journal of nursing studies. 2020;108:103632.
28. O'Connor C, Leyritana K, Doyle AM, Birdthistle I, Lewis JJ, Gill R, Salvaña EM. Delivering an mHealth adherence support intervention for patients with HIV: Mixed methods process evaluation of the Philippines connect for life study. JMIR Formative Research. 2022;6(8):e37163.
29. Sánchez SA, Ramay BM, Zook J, de Leon O, Peralta R, Juarez J, Cocohoba J. Toward improved adherence: a text message intervention in an human immunodeficiency virus pediatric clinic in Guatemala City. Medicine. 2021;100(10).
30. Mukund Bahadur K-C, Murrayb PJ. Cell phone short messaging service (SMS) for HIV/AIDS in South Africa: a literature review. MEDINFO 2010. 2010:530-4.
31. Lester R, Karanja S. Mobile phones: exceptional tools for HIV/AIDS, health, and crisis management. The Lancet infectious diseases. 2008;8(12):738-9.
32. Hlophe LD, Tamuzi JL, Shumba C, Nyasulu PS. Barriers to anti-retroviral therapy adherence among adolescents aged 10 to 19 years living with HIV in sub-Saharan Africa: A mixed-methods systematic review protocol. Plos one. 2022;17(9):e0273435.
33. Ammon N, Mason S, Corkery J. Factors impacting antiretroviral therapy adherence among human immunodeficiency virus–positive adolescents in Sub-Saharan Africa: a systematic review. Public health. 2018;157:20-31.
34. United Nations Social and Economic Council. Progress towards the Sustainable Development Goals. Report of the Secretary-General. NY. USA; 2022.
35. Creswell JW, Klassen AC, Plano Clark VL, Smith KC. Best practices for mixed methods research in the health sciences. Bethesda (Maryland): National Institutes of Health. 2011;2013:541-5.
36. Lincoln YS, Guba EG. Naturalistic inquiry: sage; 1985.
37. Schulz KF, Altman DG, Moher D. CONSORT 2010 statement: updated guidelines for reporting parallel group randomised trials. Journal of Pharmacology and pharmacotherapeutics. 2010;1(2):100-7.
38. Graneheim UH, Lundman B. Qualitative content analysis in nursing research: concepts, procedures and measures to achieve trustworthiness. Nurse education today. 2004;24(2):105-12.
39. World Medical Association. World Medical Association Declaration of Helsinki: ethical principles for medical research involving human subjects. Jama. 2013;310(20):2191-4.

Figure legend/caption

Figure 1 Flow Chart Diagram

Caption: The flowchart shows the flow diagram of mobile phone text messaging reminder in the intervention group.

For peer review only - http://bmjopen.bmj.com/site/about/guidelines.xhtml

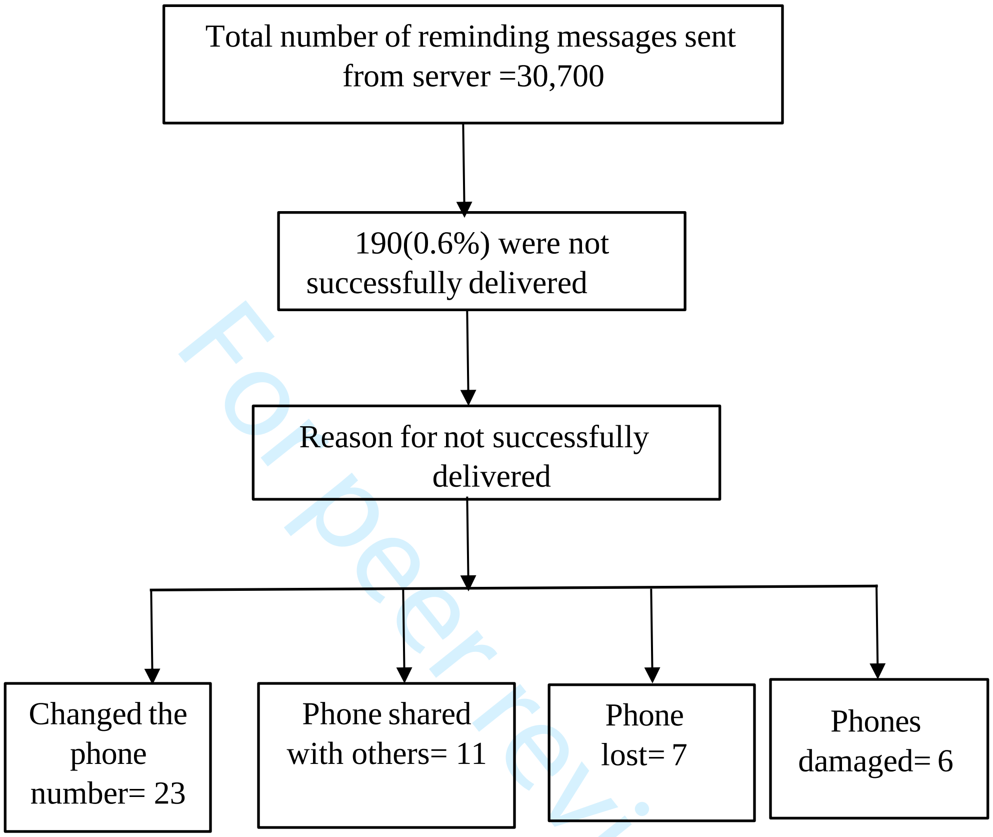

Figure 1. The flow diagram of mobile phone text messaging reminder in the intervention group

For peer review only

Semistructured interview guide

Sociodemography of participants

S.no.	Age	Sex	Educational level	Caregiver/adolescent/healthcare provider	Hospital (site)

For peer review only

Adolescents

1. Are you receiving the mobile text-message reminder from the hospital?

Probe on:

- Do you remember the code through which you receive the text-message reminder?
- What would you like about the message?
- Is there any difficulty in the use of text terms for the time schedule or messaging frequency?

2. How did you find the text message reminder?

Probe:

•Benefits

•Challenges

3. What would you like to change or improve regarding the mobile text-messaging intervention?

4. For what other purposes do you use the mobile phone provided by the hospital?

5. Was there any technical problems regarding receiving text messages?

Probe: damage to devices, battery failure, text compatibility with phone,

6. How do you compare the text message reminder for medication against preintervention period?

Parents/caregivers:

1. How do family- and social-related factors affect ART adherence in adolescents?

2.What is the role of parents/caregivers in preventing adolescent antiretroviral treatment fatigue?

3.Have you heard about the text message intervention that your child receives?

4. How happy are you of the text-message reminder intervention?

5. Do you think that sending text-message reminders supports adherence?

6. Are the adolescent properly using the mobile phone provided from hospital? (probe: damage, stolen, used by other than the adolescent)

Health care providers

1. How did you experience the communication with the adolescents regarding this intervention?

2. Do you think this intervention improves the retention and adherence of adolescents?

3. Reporting damaged equipment

4. Benefits, challenges, and hope to integrate with the health care system?

For peer review only
